# Supplementary material for: Variations in echolocation click characteristics of finless porpoise in response to day/night and absence/presence of vessel noise
Source: PLoS One. 2023 Aug 4;18(8):e0288513. doi: 10.1371/journal.pone.0288513 (PMC10403093; doi:10.1371/journal.pone.0288513)
Supplement: S1 Table — The explanatory variables were listed in descending order of the absolute values of their estimates, except for Intercept. (DOCX) [file pone.0288513.s003.docx]

**Supporting Information For**

**Variations in echolocation click characteristics of finless porpoise in response to day/night and absence/presence of vessel noise**

Mayu Ogawa^1,2*^, Satoko S. Kimura^1,2,3^

**S1 Table. Results of the best fitting GLMs analysis for several echolocation characteristics.** The explanatory variables were listed in descending order of the absolute values of their estimates, except for Intercept.

| **Fixed effect** | **Estimate** | **Std. Errod** | ***t* value** | ***p* value** |
| --- | --- | --- | --- | --- |
| **ASL** |  |  |  |  |
| (Intercept) | 163.4025 | 2.7850 | 58.671 | <2e-06* |
| presence of vessel noise:day | -7.5352 | 2.2996 | -3.277 | 0.00117 |
| day | 5.8820 | 1.8304 | 3.213 | 0.00145 |
| site | 5.0336 | 1.2233 | 4.115 | 4.97e-05 |
| temperature | 0.3335 | 0.1079 | 3.091 | 0.00218 |
| presence of vessel noise | -0.1160 | 1.4221 | -0.082 | 0.93504 |
| **peak frequency** |  |  |  |  |
| (Intercept) | 7.446e-03 | 1.922e-05 | 387.4 | <2e-16 |
| **center frequency** |  |  |  |  |
| (Intercept) | 5.477e-03 | 8.436e-04 | 6.493 | 3.33e-10 |
| day | -4.688e-05 | 2.858e-05 | -1.640 | 0.101933 |
| presence of vessel noise | -4.662e-05 | 3.000e-05 | -1.554 | 0.121164 |
| noise level | 1.549e-05 | 7.238e-06 | 2.140 | 0.033122 |
| temperature | 1.029e-05 | 2.651e-06 | 3.881 | 0.000127 |
| **-3dB BW** |  |  |  |  |
| (Intercept) | 0.1074133 | 0.0379332 | 2.832 | 0.00493 |
| day | 0.0076502 | 0.0015426 | 4.959 | 1.17e-06 |
| site | -0.0048200 | 0.0017109 | -2.817 | 0.00516 |
| temperature | 0.0009186 | 0.0001245 | 7.379 | 1.47e-12 |
| noise level | -0.0007359 | 0.0003281 | -2.243 | 0.02560 |
| **click duration** |  |  |  |  |
| (Intercept) | -2.409e-03 | 1.094e-02 | -0.220 | 0.826 |
| site | 2.460e-03 | 4.127e-04 | 5.961 | 6.80e-09 |
| day | -2.030e-03 | 3.508e-04 | -5.788 | 1.74e-08 |
| noise level | 1.395e-04 | 9.540e-04 | 1.462 | 0.145 |
| temperature | 5.131e-05 | 3.357e-05 | 1.529 | 0.127 |
| **ICI** |  |  |  |  |
| (Intercept) | -0.116320 | 0.040076 | -2.903 | 0.003965 |
| day | -0.008266 | 0.001328 | -6.222 | 1.57e-09 |
| noise level | 0.001242 | 0.000344 | 3.609 | 0.000357 |
| **number of clicks per train** |  |  |  |  |
| (Intercept) | -0.2554987 | 0.1350694 | -1.892 | 0.05947 |
| day | -0.0054758 | 0.0040273 | -1.360 | 0.17491 |
| noise level | 0.0028328 | 0.0011580 | 2.446 | 0.01498 |
| temperature | -0.0011949 | 0.0004226 | -2.828 | 0.01498 |
