# Supplementary material for: Variations in echolocation click characteristics of finless porpoise in response to day/night and absence/presence of vessel noise
Source: PLoS One. 2023 Aug 4;18(8):e0288513. doi: 10.1371/journal.pone.0288513 (PMC10403093; doi:10.1371/journal.pone.0288513)
Supplement: Attachment — Submitted filename: PONE-D-22-11363_Rev.pdf [file pone.0288513.s004.pdf]

PLOS ONE

Changes in finless porpoise echolocation clicks in response to diel and vessel traffic presence

--Manuscript Draft--

**Manuscript Number:**	PONE-D-22-11363
**Article Type:**	Research Article
**Full Title:**	Changes in finless porpoise echolocation clicks in response to diel and vessel traffic presence
**Short Title:**	Changes in echolocation click of finless porpoise
**Corresponding Author:**	Mayu Ogawa<br>Kyoto University<br>Kyoto, JAPAN
**Keywords:**	marine mammal; cetacean; odontocete; dolphin; vocalization; ocean noise pollution
**Abstract:**	Small odontocetes produce echolocation clicks to navigate their environments and to find prey, making it an important role in their survival. The effect of vessel noise on animal behavior has attracted attention owing to recent increases in vessel activities. However, changes in the echolocation clicks characteristics due to the surrounding environment have not been well-studied. In this study, on-axis echolocation clicks of free-ranging narrow-ridge finless porpoises (Neophocaena asiaeorientalis sunameri) were examined at two different sites in the wild to reveal the effects of diel response and presence of vessels on the click characteristics. The length of echolocation clicks was found to be shorter at night regardless of the diel cycle of visitation or feeding sound rate. The effects of vessel traffic varied depending on the observation sites, and responses were not constant for the same species. At the area with less vessel traffic, there was a significant decrease in the source level of echolocation clicks and an increase in the number of pulses per a click train when in the minute before and after vessel traffic noise. In contrast, at the other area with more vessel traffic, there were decreases in click length and the number of cycles per a click and an increase in the source level. Furthermore, there were significant differences in click length, bandwidth, number of cycles in a click, and number of pulses in a click train between the two sites. However, differences in source level, inter-click interval, and frequency were not significant between the sites. The findings of this study indicate that finless porpoises regulate their characteristics of echolocation clicks in response to diel change and presence of vessels, primarily by regulating click length in an echolocation click.
**Order of Authors:**	Mayu Ogawa
	Satoko Soen Kimura, PhD.
**Additional Information:**	
**Question**	**Response**
**Financial Disclosure**<br><br>Enter a financial disclosure statement that describes the sources of funding for the work included in this submission. Review the submission guidelines for detailed requirements. View published research articles from *PLOS ONE* for specific examples.<br><br>This statement is required for submission and **will appear in the published article** if the submission is accepted. Please make sure it is accurate.	This work was supported by SPIRITS 2020 of Kyoto University, JSPS KAKENHI, Grant Number JP19K20460, the Sasakawa Scientific Research Grant from The Japan Science Society, and JST FOREST Program, Grant Number JPMJFR2171. The funders had no role in study design, data collection and analysis, decision to publish, or preparation of the manuscript.

Powered by Editorial Manager® and ProduXion Manager® from Aries Systems Corporation

**Unfunded studies**<br>Enter: *The author(s) received no specific funding for this work.*<br><br>**Funded studies**<br>Enter a statement with the following details:<br>• Initials of the authors who received each award<br>• Grant numbers awarded to each author<br>• The full name of each funder<br>• URL of each funder website<br>• Did the sponsors or funders play any role in the study design, data collection and analysis, decision to publish, or preparation of the manuscript?<br>• **NO** - Include this sentence at the end of your statement: *The funders had no role in study design, data collection and analysis, decision to publish, or preparation of the manuscript.*<br>• **YES** - Specify the role(s) played.<br><br>* typeset	
**Competing Interests**<br><br>Use the instructions below to enter a competing interest statement for this submission. On behalf of all authors, disclose any competing interests that could be perceived to bias this work—acknowledging all financial support and any other relevant financial or non-financial competing interests.<br><br>This statement is required for submission and **will appear in the published article** if the submission is accepted. Please make sure it is accurate and that any funding sources listed in your Funding Information later in the submission form are also declared in your Financial Disclosure statement.<br><br>View published research articles from *PLOS ONE* for specific examples.	The authors have declared that no competing interests exist.

**NO authors have competing interests**<br><br>Enter: *The authors have declared that no competing interests exist.*<br><br>**Authors with competing interests**<br><br>Enter competing interest details beginning with this statement:<br><br>*I have read the journal's policy and the authors of this manuscript have the following competing interests: [insert competing interests here]*<br><br>* typeset	
**Ethics Statement**<br><br>Enter an ethics statement for this submission. This statement is required if the study involved:<br><br>• Human participants<br>• Human specimens or tissue<br>• Vertebrate animals or cephalopods<br>• Vertebrate embryos or tissues<br>• Field research<br><br>Write "N/A" if the submission does not require an ethics statement.<br><br>General guidance is provided below. Consult the submission guidelines for detailed instructions. **Make sure that all information entered here is included in the Methods section of the manuscript.**	The protocol of this study was non-invasive and approved by the Animal Experiment Committee of Kyoto University (Inf-K21008). All observations were deployed under the control of local fisheries association.

Powered by Editorial Manager® and ProduXion Manager® from Aries Systems Corporation

**Format for specific study types**<br><br>**Human Subject Research (involving human participants and/or tissue)**<br>• Give the name of the institutional review board or ethics committee that approved the study<br>• Include the approval number and/or a statement indicating approval of this research<br>• Indicate the form of consent obtained (written/oral) or the reason that consent was not obtained (e.g. the data were analyzed anonymously)<br><br>**Animal Research (involving vertebrate animals, embryos or tissues)**<br>• Provide the name of the Institutional Animal Care and Use Committee (IACUC) or other relevant ethics board that reviewed the study protocol, and indicate whether they approved this research or granted a formal waiver of ethical approval<br>• Include an approval number if one was obtained<br>• If the study involved *non-human primates*, add *additional details* about animal welfare and steps taken to ameliorate suffering<br>• If anesthesia, euthanasia, or any kind of animal sacrifice is part of the study, include briefly which substances and/or methods were applied<br><br>**Field Research**<br><br>Include the following details if this study involves the collection of plant, animal, or other materials from a natural setting:<br>• Field permit number<br>• Name of the institution or relevant body that granted permission	
**Data Availability**<br><br>Authors are required to make all data underlying the findings described fully available, without restriction, and from the time of publication. PLOS allows rare exceptions to address legal and ethical concerns. See the PLOS Data Policy and FAQ for detailed information.	Yes - all data are fully available without restriction

A Data Availability Statement describing where the data can be found is required at submission. Your answers to this question constitute the Data Availability Statement and **will be published in the article**, if accepted.<br><br>**Important:** Stating 'data available on request from the author' is not sufficient. If your data are only available upon request, select 'No' for the first question and explain your exceptional situation in the text box.<br><br>Do the authors confirm that all data underlying the findings described in their manuscript are fully available without restriction?	
**Describe where the data may be found in full sentences. If you are copying our sample text, replace any instances of XXX with the appropriate details.**<br><br>• If the data are **held or will be held in a public repository**, include URLs, accession numbers or DOIs. If this information will only be available after acceptance, indicate this by ticking the box below. For example: *All XXX files are available from the XXX database (accession number(s) XXX, XXX.).*<br>• If the data are all contained **within the manuscript and/or Supporting Information files**, enter the following: *All relevant data are within the manuscript and its Supporting Information files.*<br>• If neither of these applies but you are able to provide **details of access elsewhere**, with or without limitations, please do so. For example:<br><br>*Data cannot be shared publicly because of [XXX]. Data are available from the XXX Institutional Data Access / Ethics Committee (contact via XXX) for researchers who meet the criteria for access to confidential data.*<br><br>*The data underlying the results presented in the study are available from (include the name of the third party*	All relevant data are within the manuscript and its Supporting Information files.

and contact information or URL).<br>• This text is appropriate if the data are owned by a third party and authors do not have permission to share the data.<br><br>* typeset	
Additional data availability information:	

Powered by Editorial Manager® and ProduXion Manager® from Aries Systems Corporation

Manuscript

Click here to access/download;Manuscript;Manuscript_PLOSONE_OgawaM

TITLE PAGE

Changes in finless porpoise echolocation clicks in response to diel and vessel traffic presence

Mayu Ogawa[1,2*], Satoko S. Kimura[1,2,3]

[1]Graduate School of Agriculture, Kyoto University, Kyoto, Japan

[2]Distinguished Doctoral Program of Platforms, Center for Educational Program Promotion in Graduate School, Kyoto University, Kyoto, Japan

[3]Center for Southeast Asian Studies, Kyoto University, Kyoto, Japan

*Corresponding author

E-mail: ogawa.mayu.47m@st.kyoto-u.ac.jp

Short title: Changes in echolocation click of finless porpoise

Abstract

Small odontocetes produce echolocation clicks to navigate their environments and to find prey, making it an important role in their survival. The effect of vessel noise on animal behavior has attracted attention owing to recent increases in vessel activities. However, changes in the echolocation clicks characteristics due to the surrounding environment have not been well-studied. In this study, on-axis echolocation clicks of free-ranging narrow-ridge finless porpoises (*Neophocaena asiaeorientalis sunameri*) were examined at two different sites in the wild to reveal the effects of diel response and presence of vessels on the click characteristics. The length of echolocation clicks was found to be shorter at night regardless of the diel cycle of visitation or feeding sound rate. The effects of vessel traffic varied depending on the observation sites, and responses were not constant for the same species. At the area with less vessel traffic, there was a significant decrease in the source level of echolocation clicks and an increase in the number of pulses per a click train when in the minute before and after vessel traffic noise. In contrast, at the other area with more vessel traffic, there were decreases in click length and the number of cycles per a click and an increase in the source level. Furthermore, there were significant differences in click length, bandwidth, number of cycles in a click, and number of pulses in a click train between the two sites. However, differences in source level, inter-click interval, and frequency were not significant between the sites. The findings of this study indicate that finless porpoises regulate their characteristics of echolocation clicks in response to diel change and presence of vessels, primarily by regulating click length in an echolocation click.

Introduction

Small odontocetes rely primarily on echolocation clicks for navigation and feeding [1]. The ultrasonic click sounds, are highly directional, and have a narrow beamwidth, making it difficult to accurately measure the characteristics of echolocation clicks [2]. Recently, with the establishment of observation methods [2] and improvements to the accuracy of hydrophones, the acoustic characteristics of echolocation clicks have been measured both in captivity [3–7] and in the wild [6–16]. However, in most cases, acoustic observations are conducted at the same time as visual observations from a survey vessel only during daytime [7–15]. Therefore, it is not clear how click characteristics change depending on day and night, and with the absence and presence of vessels [17]. Many animals, both marine and terrestrial, exhibit diel behavior [18–22], which has been extensively examined in odontocetes using passive acoustic monitoring (PAM) and biologging tags [17,23–29]. Diel changes in echolocation click characteristics have been observed in some species; for instance, melon-headed whales (*Peponocephala electra*) exhibit higher center frequencies at night owing to increased feeding activity [17] and harbor porpoises (*Phocoena phocoena*) exhibit longer inter-click intervals (ICI) at night to navigate at greater distances [23]. Additionally, the diel behavior of harbor porpoises likely depends on diel behavior changes in their prey [24,30]. According to previous studies, various factors (e.g., diel changes in prey species [24,30], and light conditions [23]) may change the characteristics of echolocation clicks during day and night. However, since it has not been investigated whether echolocation clicks change during day and night in many species, it is necessary to examine how other species adapt to environmental changes during day and night in order to understand the basic ecology of the species. Moreover, echolocation clicks could also be influenced by surrounding environmental factors, such as vessel noise.

Recently, the impact of vessel noise on the behavior and vocalization of aquatic species has been a concern [31–36]. Atlantic bottlenose dolphins (*Tursiops truncatus*) and harbor porpoises decreased emitting buzz (feeding trial sound [37,38]) when vessels were traveling in the nearby area [39,40], indicating that the percentage of their feeding activity decreased. Additionaly, changes in the energy budget of bottlenose dolphins were influenced by vessels [41]. Lahille's bottlenose dolphin (*T. t. gephyreus*) has been reported to emit significantly fewer echolocation clicks when vessels are present within a radius of approximately 250 m [42]. Melon-headed whales increased the source level of echolocation clicks in correlation with the ambient sound pressure level [17]. However, there is still a knowledge gap on the echolocation click characteristics of narrow-ridged finless porpoises (*Neophocaena asiaeorientalis*). The impacts of vessels on odontocetes vary among species [43] and understanding these impacts are necessary for the conservation of many other species including the narrow-ridged finless porpoise.

Narrow-ridged finless porpoises are small odontocetes found in the coastal waters of East Asia; there are two subspecies, *N. a. sunameri* found in ocean water (hereafter, referred to as finless porpoise) and *N. a. asiaeorientalis* (hereafter, referred to as Yangtze finless porpoise) found mainly in freshwater [44]. The finless porpoise is often found in sandy areas shallower than 50 m, exposing them to human activities and anthropogenic impacts, such as vessel traffic [45]. Increases in vessel traffic and noise have been shown to affect the distribution of Yangtze finless porpoise [46,47] and elevate cortisol levels [48]. Finless porpoises avoid passing boats by diving [49]. However, the effect of day and night, and presence of vessels in the sound characteristics of echolocation clicks by finless porpoise has not been investigated.

Therefore, this study aimed to measure on-axis echolocation clicks at two sites in Japan to examine the effects of day and night, and presence of vessels on the sound

characteristics of finless porpoise. Two observation sites were in waters containing genetically and morphologically different local populations of finless porpoises [50–52], with different levels of vessel traffic. Although echolocation clicks of wild finless porpoise have been reported in the coastal waters of mainland China, Liao-dong-wan Bay in the Bohai Sea, and the western coast of the Taiwan Strait [10,15], this is the first study to examine echolocation click characteristics of free-ranging finless porpoises in coastal areas of Japanese waters. The results of this study provide valuable information for conservation of this species.

Materials and methods

Study area and recording system

Acoustic observations were conducted at two sites in Japan, one in the Seto Inland Sea (St. S) and the other in Mikawa Bay (St. M) [Fig. 1, Table 1]. The St. S finless porpoise is a part of the Inland Sea-Hibiki Nada population, whereas the St. M finless porpoise belongs to the Ise-Mikawa Bay population [52]. Both observation sites had a sandy bottom sediment and fishing boat were the dominant vessels in these areas. A total of 114 fishing boats were identified in the nearby fishing ports of St. S (2018, http://www.machimura.maff.go.jp/machi/contents/35/341/details.html) and a total of 453 at St. M (2017, https://www.himaka.net/about). Additionally, ferry routes near the observation site of St. M includes more than 30 trips per day.

The protocol of this study was non-invasive and approved by the Animal Experiment Committee of Kyoto University (Inf-K21008).

Fig. 1. Observation sites and acoustic array system. (A) Distribution of finless porpoise in the seas around Japan (blue area). (B, C) Maps of St. S and St. M, with the dotted line denoting the ferry route. (D) Location of acoustic instruments. From the top, the sensors (black in the figure) were placed at 2 m (INFINITY-EM), 3 m (A-tag), 3.5 m (SoundTrap), and 4 m (A-tag) from the water surface (blue line).

Table 1. Location and device information for the acoustic observation of finless porpoise at each site in 2021.

Observation site	GPS position	Bottom depth	Total observation period	Recording duration	SoundTrap Sensitivity
**St. S**	33°51.151N, 132°06.893E	16 m	6 July 2021–6 September 2021	638 h	175.9 dB (± 3 dB)
**St. M**	34°41.833N, 136°59.555E	15 m	16 June 2021–19 September 2021	760 h	174.9 dB (± 3 dB)

Acoustic data at each station were recorded using two A-tags (ML 200-AS8, MMT, Saitama, Japan), SoundTrap 300HF (Ocean Instruments, Auckland, New Zealand), and INFINITY-EM current meters (JFE Advantech, Hyogo, Japan). An acoustic array was assembled in a vertical position, with an INFINITY-EM at a depth of 2 m, an A-tag at 3 m, a SoundTrap at 3.5 m, and an A-tag at 4 m (Fig. 1D) with buoys attached at the surface. At both sites, the acoustic arrays were placed on buoys, which were deployed under the control of local fisheries association, and acoustic data were obtained by multiple continuous acoustic observations for approximately six days.

The A-tag is a stereo event recorder of pulse information; it records the time, sound pressure level of each of two hydrophones, and the relative azimuth of a pulsed sound that exceeds a set sound pressure threshold. The A-tag was not possible to record the frequency information, but it was possible to detect the click train using the features of smooth changes in sound pressure and ICI in the click train of the echolocation [53–55]. The two hydrophones

of the A-tag have different frequency responses (maximum sensitivities at 130 and 70 kHz) and were placed approximately 190 mm apart. The time difference between the arrival of the sound from the two hydrophones of the A-tag was measured the relative azimuth, and the sound pressure ratio was discriminated between Delphinidae and Phocoenidae [56,57]. The distance to the sound source from the array was calculated using the relative azimuth of the sound source from the two A-tags and a trigonometric function [14,58]. The bandpass filter in the A-tags was adjusted to 55–235 kHz, and amplification was modified to +60 dB. The sampling rate was set to 0.25 ms and the amplitude threshold to 360 counts (approximately 6.3 Pa).

The SoundTrap 300HF was used to obtain sound information, such as the frequency and sound pressure. The sampling frequency was 576 kHz, the high-pass filter was 600 Hz, the clip levels were set to 172 dB re 1 μPa, and the self-noise was 37 dB re 1 μPa (above 2 kHz), with 16-bit resolution. The frequency response was flat in the range of 20–150 kHz, 175 dB re 1 μPa (±3 dB) at St. S, and 174.9 dB re 1 μPa (±3 dB) at St. M (Table 1).

The INFINITY-EM records the water temperature and tidal current directional velocities. This study used water temperature data to calculate sound velocity, and used synthetic flow velocities to one of the environment factors. The data were recorded in burst mode, which records 10 times at 0.1 s intervals once every 5 min. Unfortunately, due to the instrumental errors, data in St. S were not detected from July 6–12.

Data analysis

The finless porpoise echolocation clicks were screened using A-tag data. On-axis sounds were examined using A-tag and SoundTrap data, and the source parameters of echolocation clicks were measured using SoundTrap data. A-tag data were analyzed using Igor Pro 8.03 (WaveMetrics, Portland, USA), and SoundTrap data were analyzed using both

Adobe Audition 14.4 (Adobe, San Jose, USA) and MATLAB R2021a (MathWorks, Natick, USA).

First, the echolocation click trains of the finless porpoise were detected from A-tag data using Igor Pro. Selected echolocation click trains by finless porpoise were filtered using the following parameters: 1 ms or less was discarded as a reflected wave, the duration of a series of click trains was set to 1–200 ms, and the number of pulses was set to ≥ 6 [53]. According to Kameyama et al. [56] and Kimura et al. [57], when the sound pressure ratio of two hydrophones in an A-tag is set to ≥ 0.6, it can detect the sounds of most of Phocoenidae, including the finless porpoise. The echolocation click trains detected using the above filters were manually checked, referring to Akamatsu et al. [53,54] and Kimura et al. [55].

Next, time information of the echolocation clicks of the finless porpoise detected by the A-tag was referenced to filter out echolocation clicks from SoundTrap data using Adobe Audition. On-axis echolocation clicks were filtered from the A-tag and SoundTrap data using seven criteria determined with reference to previous studies [11,12,59]: (1) click trains must be measured by all five hydrophones; (2) the relative azimuth was calculated from the arrival distance measured by the A-tag must be within −35.5° to 35.5°; (3) the series of click trains had amplitudes that first increased and then decreased; (4) the maximum amplitude of the series of click trains; (5) the maximum amplitude of the clicks must be higher than the reflected wave from the sea surface or seabed; (6) the on-axis sound was visually checked and there was no distortion; and (7) the distance between the estimated source and the acoustic array was within 60 m. Analysis was conducted only when the signal-to-noise ratio (peak-to-peak amplitude) was greater than 20 dB. Criterion (1) was analyzed using data from A-tag and SoundTrap, (2) and (7) using data from A-tag, and (3) to (6) using data from SoundTrap. The distance between the finless porpoise and acoustic array was estimated using the equation of Kimura et al. [14,58].

Finally, the source parameters for the on-axis echolocation clicks of finless porpoise, including apparent source level (ASL, dB re 1 μPa), ICI (ms), peak frequency (kHz), center frequency (kHz), t (μs), −3 dB bandwidth (BW, kHz), number of cycles, and number of pulses, were calculated using MATLAB. ASL was calculated using equation (1) by Mohl et al. [60]:

$$ASL = RL + TL, \quad (1)$$

where *RL* is the received level and *TL* is the transmission loss, which was calculated using equation (2):

$$TL = 20\log r + r\alpha, \quad (2)$$

where r is the estimated distance from the finless porpoise to the acoustic array and α is calculated using the Francois and Garrison equation [61]. The water temperature data used for the calculation of α were obtained from INFINITY-EM, and data from July 6–12, when St. S could not be recorded, were obtained from the Hirae Iwakuni fixed-line survey conducted by the Inland Sea Research Department of the Yamaguchi Fisheries Research Center during the same period (https://www.pref.yamaguchi.lg.jp/cms/a16403/uminari_network/suion_enbun.html). The salinity data for St. S were obtained from the same temperature data for St. S. Salinity data for St. M were obtained from the automatic sea condition monitoring buoy in Mikawa Bay No. 2 (34°44’42″N, 137°04’19″E), available at the Aichi Fisheries Research Institute (http://suisanshiken-buoy.jp/top/index.html). The α was calculated using variable values of water temperature and salinity, and was 0.044 ± 0.004 dB/m (average ± SD). The ICI was calculated average sound interval of the pulses before and after the maximum amplitude of the series of click train. The frequency of the maximum amplitude was defined as the peak frequency, the average of frequencies at −3 dB from the amplitude of the peak frequency was defined as the center frequency, and the frequency bandwidth was defined as −3 dB BW. The

t refers to the time from when the amplitude of the click becomes higher than the background noise to when it became smaller than the background noise. The number of cycles was defined as the number of cycles included in one click, and the number of pulses indicated the number of pulses included in the click train.

The detected sounds were categorized into day and night based on the time of detection and sunset information of each area. For sunset information, the data of Sunrise Sunset Times Lookup provided by MAPLOGS.COM was referenced (St. S: https://sunrise.maplogs.com/ja/nagashima_kaminoseki_ch_kumage_gun_yamaguchi_ken_japan.72598.html, St. M: https://sunrise.maplogs.com/ja/higashigawa_himakajima_minamichita_ch_chita_gun_aichi_ken_japan.78580.html). The absence and presence of vessels was determined by manual inspection of the sounds 1 min before and after the on-axis echolocation click. The root mean square (rms) amplitudes at 1 min before and after each echolocation clicks were calculated using the rms function of MATLAB and defined as the noise level (dB re 1 μPa).

All statistical analyses were conducted using the R software (version 3.6.3, R Core Team, 2020). The echolocation click source parameters, diel response and effects of vessel presence at each site were compared using a Student’s t-test or Mann–Whitney U-test. The Pearson correlation coefficient and Kendall rank correlation coefficient were analyzed to determine the relationships among all source parameters. Additionally, the relationships with all source parameters between water temperature, salinity, synthetic flow velocity, and noise level were determined using Kendall rank correlation coefficients.

Based on data obtained from the A-tag placed at a depth of 4 m, the rate of echolocation clicks (on-axis and off-axis) encountered by the finless porpoise was calculated. Click trains with an average ICI <10 ms were calculated separately from regular clicks as

buzz [37,38,55,62]. All data were pooled, and the ratio of buzz to the total number of click trains was calculated for day and night, which were 6:00–17:59 and 18:00–5:59, respectively.

Results

A total of 64 on-axis echolocation clicks were detected during approximately 638 h of recordings at St. S and 74 clicks during 757 h of recording at St. M (Fig. 2, Table 2). During daytime, 40 on-axis echolocation clicks were detected at St. S and 9 clicks at St. M, whereas during nighttime, a total of 24 echolocation clicks were detected at St. S and 65 clicks at St. M. In the absence of vessels, the numbers of on-axis echolocation clicks were 39 at St. S and 24 at St. M, while the numbers in the presence of vessel sounds were 25 at St. S and 37 at St. M. The ASL of the Japanese coastal finless porpoise was 179 ± 8 dB re 1μPa (average ± SD) at the two sites, 178 ± 8 dB re 1μPa at St. S, and 180 ± 7 dB re 1μPa at St. M (Table 2). The average peak frequency at both sites was 134 ± 6 kHz, 133 ± 6 kHz at St. S and 135 ± 6 kHz at St. M (Table 2). The echolocation clicks was concentrated in the ultrasonic band, so there was no masking by noise such as ship noise. Additionally, there were significant differences in t, −3 dB BW, number of cycles, and number of pulses between St. S and St. M (Table 2).

Fig. 2. Typical echolocation clicks of finless porpoise. (a) Waveforms and (b) signal frequency. Both (a) and (b) are sounds obtained from the St. S data.

Table 2. Summary of finless porpoise echolocation clicks parameters at two observation points. ASL and peak frequency were compared using the Student's t-test, and other parameters were compared using Mann–Whitney U-test to determine significant differences between sites. In this manuscript, $p < 0.05$ is shown as > or < and $p < 0.01$ as >> or <<; > and

>> indicate that the source parameter in St. S was larger than St. M, while < and << indicate that the source parameter in St. M was larger than St. S.

	Total		St. S			St. M	
**Source parameter**	Average ± standard deviation (SD)	Range	Average ± SD	Range	p	Average ± SD	Range
**ASL (dB re 1 μ Pa)**	179±8	160–198	178±8	165–198	0.544	180±7	160–197
**ICI (ms)**	40±17	8–127	41±22	8–127	0.996	37±11	10–77
**peak frequency (kHz)**	134±6	120–146	133±6	121–144	0.095	135±6	120–146
**center frequency (kHz)**	133±3	120–141	133±3	126–141	0.928	133±3	120–140
**t (μ s)**	66±13	42–116	73±14	45–116	>>	61±8	42–82
**−3 dB BW (kHz)**	19±5	7–34	17±4	7–33	<<	21±4	11–34
**number of cycle**	9±2	6–15	10±2	7–15	>>	8±1	6–11
**number of pulse**	26±21	6–137	30±19	7–72	>>	23±22	6–137
**range from array (m)**	23±11	6–59	25±11	10–56	-	22±10	6–59

Abbreviations: ASL, apparent source level; ICI, inter-click interval; t, time of a pulse; BW, bandwidth.

Compared to daytime, there were significant decreases in t (U-test, p = 0.005; Fig. 3d) and number of cycles (U-test, p = 0.002; Fig. 3f), but a significant increase in −3 dB BW (U-test, p = 0.021; Fig. 3e) at night in St. S. However, there were no significant changes in ASL (t-test, p = 0.586; Fig. 3a), ICI (U-test, p = 0.765; Fig. 3b), peak frequency (t-test, p = 0.858; Fig. 3c), center frequency (t-test, p = 0.747), or number of pulses (U-test, p = 0.593; Fig. 3g). In St. M, there were significant decreases in t (U-test, p < 0.001; Fig. 3d) and the number of cycles (U-test, p = 0.018 Fig. 3f) at night compared with daytime. However, there were no significant changes in ASL (t-test, p = 0.753; Fig. 3a), ICI (U-test, p = 0.700; Fig. 3b), peak frequency (t-test, p = 0.427; Fig. 3c), center frequency (U-test, p = 0.591), −3 dB BW (t-test,

p = 0.334; Fig. 3e), or number of pulses (U-test, p = 0.422; Fig. 3g). Regarding the effect of vessel traffic, there was a significant decrease in ASL (t-test, p = 0.003; Fig. 3a) and a significant increase in the number of pulses (U-test, p = 0.001; Fig. 3g) when vessels were present within a minute before and after compared with that in the absence of vessel traffic at St. S. However, there were no significant changes in ICI (U-test, p = 0.772; Fig. 3b), peak frequency (t-test, p = 0.785; Fig. 3c), center frequency (t-test, p = 0.112), t (U-test, p = 0.894; Fig. 3d), −3 dB BW (U-test, p = 0.635; Fig. 3e), or number of cycles (U-test, p = 0.992; Fig. 3f). In St. M, there were significant increases in t (U-test, p = 0.014; Fig. 3d) and the number of cycles (U-test, p = 0.008; Fig. 3f) when a vessel was present compared with an absence of vessel traffic. However, there were no significant changes in ASL (t-test, p = 0.651; Fig. 3a), ICI (U-test, p = 0.378; Fig. 3b), peak frequency (t-test, p = 0.823; Fig. 3c), center frequency (U-test, p = 0.301), −3 dB BW (t-test, p = 0.092; Fig. 3e), or number of pulses (U-test, p = 0.287; Fig. 3g).

Fig. 3. Effect of day and night and presence of vessels on the source parameters. (a) Apparent source level (ASL), (b) inter-click interval (ICI), (c) peak frequency, (d) time interval between when the amplitude of the click became larger than the background noise to when it became smaller than the background noise (t), (e) −3 dB bandwidth (BW), (f) number of cycles, and (g) number of pulses. D indicates as day, N indicates as night, P denotes the presence of vessels, and A denotes the absence of vessels. St. S and St. M are shown in light blue and red, respectively. The results of Student's t-test or Mann–Whitney U-test are shown as * for $p < 0.05$ and ** for $p < 0.01$.

Correlation analysis of the source parameters showed that there was a negative correlation between t and −3 dB BW (Kendall correlation coefficient = −0.523, $p < 0.001$;

Fig. 4a, S1 Table), a positive correlation between t and the number of cycles (Kendall correlation coefficient = 0.766, $p < 0.001$; Fig. 4b, S1 Table), and a negative correlation between −3 dB BW and the number of cycles (Kendall correlation coefficient = −0.629, $p < 0.001$; Fig. 4c, S1 Table). However, there were no correlations among the other source parameters (Pearson or Kendall correlation coefficients < 0.5 and > -0.5; S1 Table). Additionally, there was no significant correlation ($-0.3 <$ Kendall correlation coefficients < 0.3) between each source parameter and water temperature, salinity, synthetic flow velocity, and noise level (S2 Table).

Fig. 4. Scatter plots and regression lines. (a) t and −3 dB bandwidth (BW), (b) number of cycles and t, and (c) number of cycles and −3 dB BW.

The numbers of regular clicks and buzzes recorded were calculated for each hour at both sites (Fig. 5). A total of 913 click trains were recorded at St. S, with 64.2% (n = 586) observed during the day, and 35.8% (n = 327) observed at night. A total of 1,525 click trains were recorded at St. M, with 18.7% (n = 285) observed during the day, and 81.3% (n = 1,240) observed at night. In St. S, buzz accounted for 8.2% (n = 48) of all click trains in the daytime and 3.1% (n =10) in the nighttime. In St. M, buzz accounted for 1.4% (n = 4) of all click trains in the daytime and 2.0% (n = 25) in the nighttime. The buzz rate, which is the percentage of total buzz to total click train, was 6.4% for St. S and 1.9% for St. M.

Fig. 5. Number of regular clicks and buzzes detected in all observation periods. (a) St. S, and (b) St. M. Note that sounds include both on- and off-axis.

DISCUSSION

In this study, characteristic on-axis echolocation clicks of wild finless porpoises were measured along the coast of Japan for the first time. Subsequently, the effects of day and night and presence of vessel on the source parameters of the finless porpoise were examined at two different sites.

Effects of day and night on echolocation clicks

We observed differences in the source parameter at both sites between day and night. At St. S, the numbers of regular clicks and buzzes recorded were higher during the daytime than at night (Fig. 5a), indicating a higher presence rate during the daytime. The percentage of buzz in all click trains was higher during the daytime (8.2%) than that during the nighttime (3.1%). In St. M, the number of click trains was higher at night (Fig. 5b), indicating more presence at night. Additionally, the percentage of buzz in all click trains was low in both daytime (1.4%) and nighttime (2.0%). These results indicate that there is a difference in day and night presence rate and feeding rates between St. S and St. M; however, both sites exhibited similar trends for t, −3 dB BW, and the number of cycles, although there was a correlation between −3 dB BW, and t (Fig. 4a). These results suggest that finless porpoises exhibit a decrease in t at night, regardless of their encounter or feeding rate (Fig. 3, Fig. 5). It was hypothesized that the lesser the t, the higher the accuracy of binaural time measurements, resulting in improved localization ability. Atlantic bottlenose dolphin localize using interaural differences in the arrival time of sound, by binaural phase differences due to each ear being at a different point in the phase angle, or binaural intensity cues [63]. There is less visual information at night than during the daytime, which causes finless porpoises to rely more on acoustic information. If finless porpoises localize in the same way as Atlantic bottlenose dolphin, it might be possible for the species to obtain more accurate spatial information in the dark by regulating the t. Changes in echolocation clicks between day and night have been

reported for melon-headed whales [17]; to the best of our knowledge, this is the first study to report such changes in odontocetes. It is necessary to identify whether decreases in the click length can improve the localization ability of finless porpoises. Additionally, it is important to verify whether finless porpoises have the ability to adjust the click length. Further comprehensive studies are necessary to improve our understanding of echolocation clicks by odontocetes.

Generally, waveform energy increases with increasing sound length without changing the source level [13]. In the present study, although ASL values were similar between day and night, t was significantly lower at night, indicating that waveform energy was lower at night. Therefore, it can be inferred that the source parameter was shifted to save energy during the night. In previous studies, several odontocetes, including harbor porpoise [24] and Risso's dolphin (*Grampus griseus*) [25], have been reported to actively produce echolocation clicks and buzzes at night and was linked to active feeding at night. Previous studies have shown that the Yangtze finless porpoise more actively feeds at night [27,64]. Based on this information, it was hypothesized that finless porpoises may exhibit to use more energy for feeding activities. More energy may be required for feeding at night due to poor visual information. It seems that finless porpoises save the energy during the searching phase, when most of the time is spent, in preparation for feeding, which needs a lot of energy. Further studies on energy balance and energy cost of feeding during the day and at night are necessary to verify this hypothesis.

Harbor porpoises have been reported to increase the emission rate of echolocation clicks [65] and increase ICI at night to search for distant objects [23]. These results suggests that the vocalization characteristics of finless porpoises may similarly vary depending on the brightness of the surroundings. Nevertheless, there is still an uncertainty of the visibility

capabilities of finless porpoise. Future research on the visual dependency of finless porpoises will assist in further explanation of the day and night differences observed in this study.

Effects of presence of vessel traffic on echolocation clicks

There were considerable differences in echolocation clicks in response to the presence of vessel traffic between St. S and St. M (Fig. 3). At St. S, there was a significant decrease in ASL and a significant increase in the number of pulses when vessels were present (Fig. 3a, 3g), which is contrary to some previous findings [17,66]. For instance, it was reported that melon-headed whales increase in the source level of their echolocation clicks with increasing ambient sound levels which includes vessel noise [17]. Additionally, an increase in the source level of echolocation clicks with increasing background noise has been reported for beluga (*Delphinapterus leucas*) [66]. The tendency for the source level to increase as the ambient source level increases is known as the Lombert effect [67] and is thought to prevent the sound from being masked by ambient sounds. For the first time, our study shows that finless porpoises reduce the source level of echolocation clicks when vessels are present (within a minute before and after). Previous studies reported a decrease in the vocalization rates (including echolocation clicks and whistles) due to vessel present [43,68], and this was attributed to vigilance behavior from approaching vessels [39,43]. Beluga have been reported to emit what was known to be a warning sound when vessels were approaching [68]. These findings suggest that the decrease in ASL observed at St. S might also be due to vigilance behavior by finless porpoises in response to the approaching vessels. The increase in the number of pulses was considered to compensate for the energy generated by the decrease in source level or avoid masking by noise.

In contrast, there was an increase in ASL at St. M when a vessel was present, but it was not statistically significant. Additionally, there was a significant increase in t and the

number of cycles at St. M when a vessel was present (Fig. 3d and 3f). These changes in the source parameter increased the energy of the waveform, which may contribute to preventing the masking of echolocation clicks.

It can be hypothesized that the porpoises in St. S and St. M differed in their habituate vessels and their response to vessel traffic, which resulted response in vigilance behavior in St. S and avoidance of masking in St. M. Previous studies have shown that the rate of escape behavior from vessels varies with the condition and behavior type, habituate with the vessel, type of vessel, and speed [43,69,70]. A study has shown that the rate of the whistle, a communication sound produced by most dolphins, varied with the level of vessel traffic, with lower whistle rates when exposed to fewer vessels and a higher whistle rate when exposed to more vessels [43]. The porpoises around St. M were more exposed to more vessel noise than those in St. S because of their proximity to a ferry route and nearby fishing port. Additionally, the diel cycle of visitation and feeding events of finless porpoises differed between St. S and St. M (Fig. 5). Although the level of vessel traffic, habituate, and behavior in response to vigilance behavior and masking changes was not examined, our results show that the same species could have opposed responses to vessel traffic and noise.

The decrease in ASL observed in St. S in response to vessel traffic caused a decrease in the search distance, which may result in a decrease in feeding efficiency and the fitness of the species. Although these effects were small, long-term cumulative effects may have significant consequences for species or populations [41,76,77]. Moreover, increasing the emission rate, source level, and duration of echolocation clicks can increase the energy consumption of the odontocetes [78]. In the long term, both St. S and St. M finless porpoise survival may be negatively affected by vessels.

There were considerable differences in the effect of day and night, and presence of vessels, especially for t (Fig. 3d). The response of finless porpoises to vessel traffic was

different between the two sites, whereas the response to day and night was similar between the two sites (Fig. 3). Overall, the day and night effect on echolocation clicks was more marked than the effect of vessels and may have a greater influence on the characteristics of echolocation clicks. Regarding the minimal impacts from vessel noise, continuation of mid-to-long-term monitoring is necessary to fully understand the behavior of finless porpoise and for future impact assessment evaluation.

In this study, frequency was not significantly affected by day and night, and the presence of vessels (Fig. 3c). A previous study reported an increase in the center frequency of echolocation clicks of melon-headed whales at night [17]. Another study found an increase in the peak frequency of echolocation clicks of the same beluga individuals when they were moved to an area with high background noise [66]. The non-significant result obtained in the present our study may be due to the inability of changing the high frequency echolocation clicks or the relationship between the frequency component of the noise source and sound frequency. The peak frequency range of echolocation clicks in melon-headed whales has been reported as 24.4–29.7 kHz [71], and beluga echolocation click energy was shown to concentrate in the 30–50 kHz range [72]. In contrast, the peak frequency range of echolocation clicks observed in this study was 120–140 kHz (Table 2). Vessel noise mainly comprises low frequencies [73–75], and the sound pressure decreases at higher frequencies. For example, when a high-frequency shift is applied to the low-frequency band, the sound pressure is rapidly reduced, and the masking effect can be effectively mitigated. However, in the ultrasonic range used by finless porpoises, even if a frequency shift occurs, there is little change in the sound pressure of the surrounding noise. Further studies are needed to verify whether the difference frequency band of echolocation clicks of other odontocetes can cause frequency shifts in vessel noise.

Effect of site on echolocation clicks

Source parameters, including t, −3 dB BW, number of cycles, and number of pulses, were significantly different at two observation sites; however, ASL, ICI, peak frequency, and center frequency were not significantly different between the sites (Table 2). The source parameter that varied the most between day and night, and presence of vessels in this study was t. Based on this result, it can be inferred that finless porpoises adapt to the surrounding environment (including day and night, and presence of vessels) mainly by regulating click length.

The difference in characteristics of echolocation clicks between St. S and St. M can be attributed to several factors. Fang et al. [6] suggested that finless porpoises adapt the characteristics of echolocation clicks depending on the surrounding environment. Other studies have shown that acoustic parameters of whistles change with behavior [79,80]. The buzz rate at St. S was 6.9% of all total click trains, whereas that of St. M was only 1.9%; indicating that the finless porpoises at St. S spent more time exhibiting feeding behavior than those at St. M. Most observed behaviors differed depending on the observation site, which may have been affected by the echolocation click characteristics of the predominant behavior. Additionally, differences in source parameters between species are thought to be caused by prey preferences, morphological differences, and ambient noise levels [66,81]. Although the degree of change caused by these factors may be small, it was assumed that these factors affected the change in sound characteristics between individuals. For example, the diel cycle of harbor porpoises depends on the diel cycle of their prey [23,24,30]. In this study, the different emission rates of buzz may reflect different prey preferences or different diel cycle of their prey. Moreover, the size and skull morphology of the Inland Sea-Hibiki Nada including St.S and Ise-Mikawa including St. M populations differ significantly [50,52], which may have influenced the source parameters of the species. It is necessary to compare

the source parameters of several populations under varying conditions to determine whether the differences in t, −3 dB BW, number of cycles, and number of pulses observed in this study are due to population differences, prey preferences, surrounding sound environment, or other factors.

Conclusions

This study examined the effect of day and night, and the presence of vessels on the characteristics of echolocation clicks of finless porpoises at two different sites. Regardless of the diel cycle of visitation and feeding, t decreased at night. This might be because the species adjusted their spatial resolution to compensate for visual information or reduced energy consumption at night by changing the length of the sound. The response to vessel traffic was not constant, and varied from site to site. At St. S, where the level of vessel traffic was low, there was a decrease in the source level of echolocation clicks and an increase in the number of pulses, which was considered a vigilance behavior. At St. M, where the level of vessel traffic was high, there was an increase in the t, number of cycles, and waveform energy, which was considered a masking behavior. Furthermore, the frequency parameters were not significantly affected by day and night, and presence of vessels, indicating that finless porpoises are incapable of changing the frequency of echolocation clicks or that the conditions did not induce a change in frequency. There was a significant difference in the acoustic parameters between the observation sites, especially t. This was most likely due to changes in the surrounding environment, individual behavior, prey preferences, and surrounding sound levels. Changes in t occurred throughout day and night, in the presence of vessels, and at both observation sites, suggesting that finless porpoises may adapt to their environment mainly by regulating echolocation click length. The changes of characteristics of

echolocation clicks in day and night had a stronger effect on t than the changes in presence of vessels.

However, this study has revealed that many other factors, such as the relationship between changes in t and spatial resolution, skull morphology, and vocalization mechanisms, needs to be investigated. Moreover, future studies are needed to examine the effect of behavioral changes, changes in echolocation click parameters on the energy balance of finless porpoises and the effect of vessel noise on the feeding behavior and energy balance of finless porpoises should be examined in future studies. The findings of this study contribute to our understanding of species adaptation mechanisms in response to nighttime and the effect of noise pollution on odontocetes and provides baseline information on basic ecology that is important for conservation.

Acknowledgments

Data acquisition for St. M. and St. S. was supported by Tsunemi Suzuki, Kengo Ueda, Tetsuya Kohama, and Shinichi Watanabe. We also thank Tomonari Akamatsu and the staffs at the Fisheries and Environment Oceanography Laboratory at Kyoto University for their support and cooperation. Finally, we would like to thank Editage for editing and reviewing this manuscript for English language.

References

1. Au WW. The sonar of dolphins. New York: Springer-Verlag New York Inc; 1993.
2. Madsen PT, Wahlberg M. Recording and quantification of ultrasonic echolocation clicks from free-ranging toothed whales. Deep Sea Res I. 2007;54: 1421-1444. doi: 10.1016/j.dsr.2007.04.020.
3. Au WW, Floyd RW, Penner RH, Murchison AE. Measurement of echolocation signals of the Atlantic bottlenose dolphin, Tursiops truncatus Montagu, in open waters. J Acoust Soc Am. 1974;56: 1280-1290. doi: 10.1121/1.1903419.
4. Kamminga C, Kataoka T, Engelsma FJ. Investigations oncetacean sonar VII: Underwater

sounds of *Neophocaena phocaenoides* of the Japanese coastal population. Aquat Mamm. 1986;12: 52–60.

5. Au WW, Pawloski JL, Nachtigall PE, Blonz M, Gisner RC. Echolocation signals and transmission beam pattern of a false killer whale (*Pseudorca crassidens*). J Acoust Soc Am. 1995;98: 51–59. doi: 10.1121/1.413643.
6. Fang L, Wang D, Li Y, Cheng Z, Pine MK, Wang K, et al. The source parameters of echolocation clicks from captive and free-ranging Yangtze finless porpoises (*Neophocaena asiaeorientalis asiaeorientalis*). PLOS ONE. 2015;10: e0129143. doi: 10.1371/journal.pone.0129143.
7. Niu F, Kittiwattanawong K, Wang X, Xue R, Sakornwimon W, Wu F, et al. A comparative study of echolocation parameters of wild and captive Indo-Pacific humpback dolphins (Sousa chinensis). Aquat Mamm. 2021;47: 574-584. doi: 10.1578/AM.47.6.2021.574.
8. Au WWL. Echolocation signals of wild dolphins. Acoust Phys. 2004;50: 454-462. doi: 10.1134/1.1776224.
9. Li S, Wang K, Wang D, Akamatsu T. Echolocation signals of the free-ranging Yangtze finless porpoise (*Neophocaena phocaenoides asiaeorientialis*). J Acoust Soc Am. 2005;117: 3288-3296. doi: 10.1121/1.1882945.
10. Li S, Wang D, Wang K, Akamatsu T, Ma Z, Han J. Echolocation click sounds from wild inshore finless porpoise (Neophocaena phocaenoides sunameri) with comparisons to the sonar of riverine N. p. asiaeorientalis. J Acoust Soc Am. 2007;121: 3938-3946. doi: 10.1121/1.2721658.
11. Villadsgaard A, Wahlberg M, Tougaard J. Echolocation signals of wild harbour porpoises, *Phocoena phocoena*. J Exp Biol. 2007;210: 56-64. doi: 10.1242/jeb.02618.
12. Kyhn LA, Jensen FH, Beedholm K, Tougaard J, Hansen M, Madsen PT. Echolocation in sympatric Peale's dolphins (*Lagenorhynchus australis*) and Commerson's dolphins (*Cephalorhynchus commersonii*) producing narrow-band high-frequency clicks. J Exp Biol. 2010;213: 1940-1949. doi: 10.1242/jeb.042440.
13. Morisaka T, Karczmarski L, Akamatsu T, Sakai M, Dawson S, Thornton M. Echolocation signals of Heaviside’s dolphins (*Cephalorhynchus heavisidii*). J Acoust Soc Am. 2011;129: 449-457. doi: 10.1121/1.3519401.
14. Kimura S, Akamatsu T, Fang L, Wang Z, Wang K, Wang D, et al. Apparent source level of free-ranging humpback dolphin, *Sousa chinensis*, in the South China Sea. J Mar Biol Assoc UK. 2016;96: 845-851. doi: 10.1017/S0025315414000071.
15. Song Z, Zhang Y, Wang X, Wei C, Wu F, Miao X. Vocalizations of a Wild Finless Porpoise (*Neophocaena asiaeorientalis sunmeri*) in the Western Coast of the Taiwan Strait, China. j biobased mat bioenergy;11: 45–52. doi: 10.1166/jbmb.2017.1642.
16. Malinka CE, Tønnesen P, Dunn CA, Claridge DE, Gridley T, Elwen SH, et al. Echolocation click parameters and biosonar behaviour of the dwarf sperm whale (Kogia sima). J Exp Biol. 2021;224: jeb240689. doi: 10.1242/jeb.240689.
17. Baumann-Pickering S, Roch MA, Wiggins SM, Schnitzler HU, Hildebrand JA. Acoustic behavior of melon-headed whales varies on a diel cycle. Behav Ecol Sociobiol. 2015;69: 1553-1563. doi: 10.1007/s00265-015-1967-0.
18. Pérez-Granados C, Schuchmann KL. Diel and seasonal variations of vocal behavior of the Neotropical White-Tipped Dove (Leptotila verreauxi). Diversity. 2020;12: 402. doi: 10.3390/d12100402.
19. Løkkeborg S, Fernö A. Diel activity pattern and food search behaviour in cod, Gadus morhua. Environ Biol Fishes. 1999;54: 345-353. doi: 10.1023/A:1007504712163.
20. Smith IP, Collins KJ, Jensen AC. Seasonal changes in the level and diel pattern of activity in the European lobster *Homarus gammarus*. Mar Ecol Prog Ser. 1999;186: 255-264. doi: 10.3354/meps186255.

21. Baumgartner MF, Fratantoni DM. Diel periodicity in both sei whale vocalization rates and the vertical migration of their copepod prey observed from ocean gliders. Limnol Oceanogr. 2008;53: 2197-2209. doi: 10.4319/lo.2008.53.5_part_2.2197.
22. Andrews KS, Williams GD, Farrer D, Tolimieri N, Harvey CJ, Bargmann G, et al. Diel activity patterns of sixgill sharks, *Hexanchus griseus*: the ups and downs of an apex predator. Anim Behav. 2009;78: 525-536. doi: 10.1016/j.anbehav.2009.05.027.
23. Carlström J. Diel variation in echolocation behavior of wild harbor porpoises. Marine Mammal Sci. 2005;21: 1-12. doi: 10.1111/j.1748-7692.2005.tb01204.x.
24. Todd VLG, Pearse WD, Tregenza NC, Lepper PA, Todd IB. Diel echolocation activity of harbour porpoises (*Phocoena phocoena*) around North Sea offshore gas installations. ICES J Mar Sci. 2009;66: 734-745. doi: 10.1093/icesjms/fsp035.
25. Soldevilla MS, Wiggins SM, Hildebrand JA. Spatial and temporal patterns of Risso's dolphin echolocation in the Southern California Bight. J Acoust Soc Am. 2010;127: 124-132. doi: 10.1121/1.3257586.
26. Au WWL, Giorli G, Chen J, Copeland A, Lammers M, Richlen M, et al. Nighttime foraging by deep diving echolocating odontocetes off the Hawaiian islands of Kauai and Ni'ihau as determined by passive acoustic monitors. J Acoust Soc Am. 2013;133: 3119-3127. doi: 10.1121/1.4798360.
27. Kimura S, Akamatsu T, Wang D, Li S, Wang K, Yoda K. Variation in the production rate of biosonar signals in freshwater porpoises. J Acoust Soc Am. 2013;133: 3128-3134. doi: 10.1121/1.4796129.
28. Linnenschmidt M, Teilmann J, Akamatsu T, Dietz R, Miller LA. Biosonar, dive, and foraging activity of satellite tracked harbor porpoises (Phocoena phocoena). Marine Mammal Sci. 2013;29: E77-E97. doi: 10.1111/j.1748-7692.2012.00592.x.
29. Shaff JF, Baird RW. Diel and lunar variation in diving behavior of rough-toothed dolphins (*Steno bredanensis*) off Kaua'i, Hawai'i. Marine Mammal Sci. 2021;37: 1261-1276. doi: 10.1111/mms.12811.
30. Brandt MJ, Hansen S, Diederichs A, Nehls G. Do man- made structures and water depth affect the diel rhythms in click recordings of harbor porpoises (*Phocoena phocoena*)? Marine Mammal Sci. 2014;30: 1109-1121. doi: 10.1111/mms.12112.
31. Guerra M, Dawson SM, Brough TE, Rayment WJ. Effects of boats on the surface and acoustic behaviour of an endangered population of bottlenose dolphins. Endang Species Res. 2014;24: 221-236. doi: 10.3354/esr00598.
32. Dyndo M, Wiśniewska DM, Rojano-Doñate L, Madsen PT. Harbour porpoises react to low levels of high frequency vessel noise. Sci Rep. 2015;5: 11083. doi: 10.1038/srep11083.
35. Marley SA, Salgado Kent CP, Erbe C, Parnum IM. Effects of vessel traffic and underwater noise on the movement, behaviour and vocalisations of bottlenose dolphins in an urbanised estuary. Sci Rep. 2017;7: 13437. doi: 10.1038/s41598-017-13252-z.
36. Velasquez Jimenez L, Fakan EP, McCormick MI. Vessel noise affects routine swimming and escape response of a coral reef fish. PLOS ONE. 2020;15: e0235742. doi: 10.1371/journal.pone.0235742.
37. Miller PJ, Johnson MP, Tyack PL. Sperm whale behaviour indicates the use of echolocation click buzzes 'creaks' in prey capture. Proc Biol Sci. 2004;271(1554): 2239-2247. doi: 10.1098/rspb.2004.2863.
38. Madsen PT, Johnson M, De Soto NA, Zimmer WM, Tyack P. Biosonar performance of foraging beaked whales (Mesoplodon densirostris). J Exp Biol. 2005;208: 181-194. doi: 10.1242/jeb.01327.
39. Pirotta E, Merchant ND, Thompson PM, Barton TR, Lusseau D. Quantifying the effect of boat disturbance on bottlenose dolphin foraging activity. Biol Conserv. 2015;181: 82-89. doi: 10.1016/j.biocon.2014.11.003.

40. Wisniewska DM, Johnson M, Teilmann J, Siebert U, Galatius A, Dietz R, et al. High rates of vessel noise disrupt foraging in wild harbour porpoises (*Phocoena phocoena*). Proc Biol Sci. 2018;285. doi: 10.1098/rspb.2017.2314.
41. Baş AA, Amaha Öztürk A, Öztürk B. Selection of critical habitats for bottlenose dolphins (*Tursiops truncatus*) based on behavioral data, in relation to marine traffic in the Istanbul Strait, Turkey. Marine Mammal Sci. 2015;31: 979-997. doi: 10.1111/mms.12202.
42. Dey M, Krishnaswamy J, Morisaka T, Kelkar N. Interacting effects of vessel noise and shallow river depth elevate metabolic stress in Ganges river dolphins. Sci Rep. 2019;9: 15426. doi: 10.1038/s41598-019-51664-1.
43. Pellegrini AY, Romeu B, Ingram SN, Daura-Jorge FG. Boat disturbance affects the acoustic behaviour of dolphins engaged in a rare foraging cooperation with fishers. Anim Conserv. 2021;24: 613-625. doi: 10.1111/acv.12667.
44. Wang JY, Frasier TR, Yang SC, White BN. Detecting recent speciation events: the case of the finless porpoise (genus *Neophocaena*). Heredity. 2008;101: 145-155. doi: 10.1038/hdy.2008.40.
45. Kasuya T, Yamamoto Y, Iwatsuki T. Abundance decline in the finless porpoise population in the Inland Sea of Japan. Raffles Bull Zool. 2002;50: 57-66.
46. Zhou L, Chen X, Duan P-X, Wang D, Wang Z-T, Wang K-X. Spatial–temporal variations in biosonar activity of Yangtze finless porpoise in the lower reaches of the Yangtze River and its correlation with underwater noise: are quieter non- shipping branches the remaining shelters? Aquatic Conserv: Mar Freshw Ecosyst. 2021;31: 964-978. doi: 10.1002/aqc.3535.
47. Mei Z, Han Y, Turvey ST, Liu J, Wang Z, Nabi G, et al. Mitigating the effect of shipping on freshwater cetaceans: the case study of the Yangtze finless porpoise. Biol Conserv. 2021;257. doi: 10.1016/j.biocon.2021.109132, 109132.
48. Nabi G, Hao Y, McLaughlin RW, Wang D. The possible effects of high vessel traffic on the physiological parameters of the critically endangered Yangtze Finless porpoise (*Neophocaena asiaeorientalis* ssp. *asiaeorientalis*). Front Physiol. 2018;9: 1665. doi: 10.3389/fphys.2018.01665.
49. Morimura N, Mori Y. Social responses of travelling finless porpoises to boat traffic risk in Misumi West Port, Ariake Sound, Japan. PLOS ONE. 2019;14: e0208754. doi: 10.1371/journal.pone.0208754.
50. Yoshida H, Shirakihara K, Shirakihara M, Takemura A. Geographic Variation in the Skull Morphology of the Finless Porpoise *Neophocaena phocaenoides* in Japan Waters. Fish Sci. 1995;61: 555-558. doi: 10.2331/fishsci.61.555.
51. Yoshida H, Yoshioka M, Shirakihara M, Chow S. Population structure of finless porpoises (*Neophocaena* phocaenoides) in coastal waters of Japan based on mitochondrial DNA sequences. J Mammal. 2001;82: 123-130. doi: 10.1644/1545-1542(2001)082<0123:PSOFPN>2.0.CO;2.
52. Yoshida H, Shirakihara M, Takemura A, Shirakihara K. Population structure of finless porpoises (*Neophocaena phocaenoides*) in coastal waters of Japan. Raffles Bull Zool. 2002;50: 35-42.
53. Akamatsu T, Teilmann J, Miller LA, Tougaard J, Dietz R, Wang D, et al. Comparison of echolocation behaviour between coastal and riverine porpoises. Deep Sea Res II. 2007;54: 290-297. doi: 10.1016/j.dsr2.2006.11.006.
54. Akamatsu T, Wang D, Wang K, Naito Y. Biosonar behaviour of free-ranging porpoises. Proc Biol Sci. 2005;272: 797-801. doi: 10.1098/rspb.2004.3024.
55. Kimura S, Akamatsu T, Wang K, Wang D, Li S, Dong S, et al. Comparison of stationary acoustic monitoring and visual observation of finless porpoises. J Acoust Soc Am. 2009;125: 547-553. doi: 10.1121/1.3021302.

56. Kameyama S, Akamatsu T, Dede A, Öztürk AA, Arai N. Acoustic discrimination between harbor porpoises and delphinids by using a simple two-band comparison. J Acoust Soc Am. 2014;136: 922-929. doi: 10.1121/1.4884763.
57. Kimura SS, Sagara T, Yoda K, Ponnampalam LS. Acoustic identification of the sympatric species Indo-Pacific finless porpoise and Indo-Pacific humpback dolphin: an example from Langkawi, Malaysia. Bioacoustics. 2021: 1-17. doi: 10.1080/09524622.2021.1998796.
58. Kimura S, Akamatsu T, Li S, Dong S, Wang K, Wang D, et al. Tracking trials of echolocating finless porpoise by two stationary passive acoustic monitoring systems. Proceedings of the 5th international symposium on SEASTAR2000 and Asian Bio-logging Science (The 9th SEASTAR2000 workshop); 2010. pp. 73-76.
59. Kyhn LA, Tougaard J, Jensen F, Wahlberg M, Stone G, Yoshinaga A, et al. Feeding at a high pitch: source parameters of narrow band, high-frequency clicks from echolocating off-shore hourglass dolphins and coastal Hector's dolphins. J Acoust Soc Am. 2009;125: 1783-1791. doi: 10.1121/1.3075600.
60. Møhl B, Wahlberg M, Madsen PT, Miller LA, Surlykke A. Sperm whale clicks: directionality and source level revisited. J Acoust Soc Am. 2000;107: 638-648. doi: 10.1121/1.428329.
61. Francois RE, Garrison GR. Sound absorption based on ocean measurements: Part I: Pure water and magnesium sulfate contributions. J Acoust Soc Am. 1982;72: 896-907. doi: 10.1121/1.388170.
62. Akamatsu T, Wang D, Wang K, Li S, Dong S. Scanning sonar of rolling porpoises during prey capture dives. J Exp Biol. 2010;213: 146-152. doi: 10.1242/jeb.037655.
63. Renaud DL, Popper AN. Sound localization by the bottlenose porpoise Tursiops truncatus. J Exp Biol. 1975;63: 569-585. doi: 10.1242/jeb.63.3.569.
64. Wang Z, Akamatsu T, Wang K, Wang D. The diel rhythms of biosonar behavior in the Yangtze finless porpoise (*Neophocaena asiaeorientalis asiaeorientalis*) in the port of the Yangtze River: the correlation between prey availability and boat traffic. PLOS ONE. 2014;9: e97907. doi: 10.1371/journal.pone.0097907.
65. Akamatsu T, Hatakeyama Y, Kojima T, Soeda H. The rate with which a harbor porpoise uses echolocation at night. In: Marine mammal sensory systems. Boston, Massachusetts: Springer; 1992. pp. 299-315. doi: 10.1007/978-1-4615-3406-8_22.
66. Au WWL, Carder DA, Penner RH, Scronce BL. Demonstration of adaptation in beluga whale echolocation signals. J Acoust Soc Am. 1985;77: 726-730. doi: 10.1121/1.392341.
67. Lombard E. Le signe de l'elevation de la voix. Ann Mal OREIL Larynx. 1911;37: 101-199.
68. Lesage V, Barrette C, Kingsley MCS, Sjare B. The effect of vessel noise on the vocal behavior of belugas in the St. Lawrence River estuary, Canada. Marine Mammal Sci. 1999;15: 65-84. doi: 10.1111/j.1748-7692.1999.tb00782.x.
69. Sini MI, Canning SJ, Stockin KA, Pierce GJ. Bottlenose dolphins around Aberdeen harbour, north-east Scotland: a short study of habitat utilization and the potential effects of boat traffic. J Mar Biol Assoc UK. 2005;85: 1547-1554. doi: 10.1017/S0025315405012774.
70. Ng SL, Leung S. Behavioral response of Indo-Pacific humpback dolphin (Sousa chinensis) to vessel traffic. Mar Environ Res. 2003;56: 555-567. doi: 10.1016/S0141-1136(03)00041-2.
71. Baumann-Pickering S, Wiggins SM, Hildebrand JA, Roch MA, Schnitzler HU. Discriminating features of echolocation clicks of melon-headed whales (*Peponocephala electra*), bottlenose dolphins (*Tursiops truncatus*), and Gray's spinner dolphins (*Stenella*

longirostris longirostris). J Acoust Soc Am. 2010;128: 2212-2224. doi: 10.1121/1.3479549.

72. Roy N, Simard Y, Gervaise C. 3D tracking of foraging belugas from their clicks: experiment from a coastal hydrophone array. Appl Acoust. 2010;71: 1050-1056. doi: 10.1016/j.apacoust.2010.05.008.
73. Wenz GM. Acoustic ambient noise in the ocean: spectra and sources. J Acoust Soc Am. 1962;34: 1936-1956. doi: 10.1121/1.1909155.
74. Ross D. Ship sources of ambient noise. IEEE J Oceanic Eng. 2005;30: 257-261. doi: 10.1109/JOE.2005.850879.
75. Abrahamsen K. The ship as an underwater noise source. In: Proceedings of the meetings on acoustics ECUA2012. Acoustical Society of America; 2012 (Vol. 17, No. 1, p. 070058). doi: 10.1121/1.4772953.
76. Stockin KA, Lusseau D, Binedell V, Wiseman N, Orams MB. Tourism affects the behavioural budget of the common dolphin Delphinus sp. in the Hauraki Gulf, New Zealand. Mar Ecol Prog Ser. 2008;355: 287-295. doi: 10.3354/meps07386.
77. Miller LJ, Solangi M, Kuczaj SA. Immediate response of Atlantic bottlenose dolphins to high-speed personal watercraft in the Mississippi Sound. J Mar Biol Assoc UK. 2008;88: 1139-1143. doi: 10.1017/S0025315408000908.
78. Holt MM, Noren DP, Dunkin RC, Williams TM. Vocal performance affects metabolic rate in dolphins: implications for animals communicating in noisy environments. J Exp Biol. 2015;218: 1647-1654. doi: 10.1242/jeb.122424.
79. Díaz López B. B. Whistle characteristics in free-ranging bottlenose dolphins (Tursiops truncatus) in the Mediterranean Sea: influence of behaviour. Mamm Biol. 2011;76: 180-189. doi: 10.1016/j.mambio.2010.06.006.
80. Hawkins ER, Gartside DF. Whistle emissions of Indo-Pacific bottlenose dolphins (*Tursiops aduncus*) differ with group composition and surface behaviors. Elizabeth R., and Donald F. J Acoust Soc Am. 2010;127: 2652-2663. doi: 10.1121/1.3308465.
81. Soldevilla MS, Henderson EE, Campbell GS, Wiggins SM, Hildebrand JA, Roch MA. Classification of Risso’s and Pacific white-sided dolphins using spectral properties of echolocation clicks. J Acoust Soc Am. 2008;124: 609-624. doi: 10.1121/1.2932059.

Supporting Tables

S1 Table. Pearson and Kendall correlation coefficient for apparent source level (ASL), inter-click intervals (ICI), peak frequency, background noise (t), and −3 dB bandwitch (BW)

S2 Table. Kendall rank correlation coefficient between each source parameter and water temperature, salinity, synthetic flow velocity, and noise level

Supporting Datas

S1 Data. Source parameters and surrounding environmental data for each of the on-axis echolocation clicks in St. S

S2 Data. Source parameters and surrounding environmental data for each of the on-axis echolocation clicks in St. M

Figure_1

Click here to access/download;Figure;Fig1_PLOSONE.tif

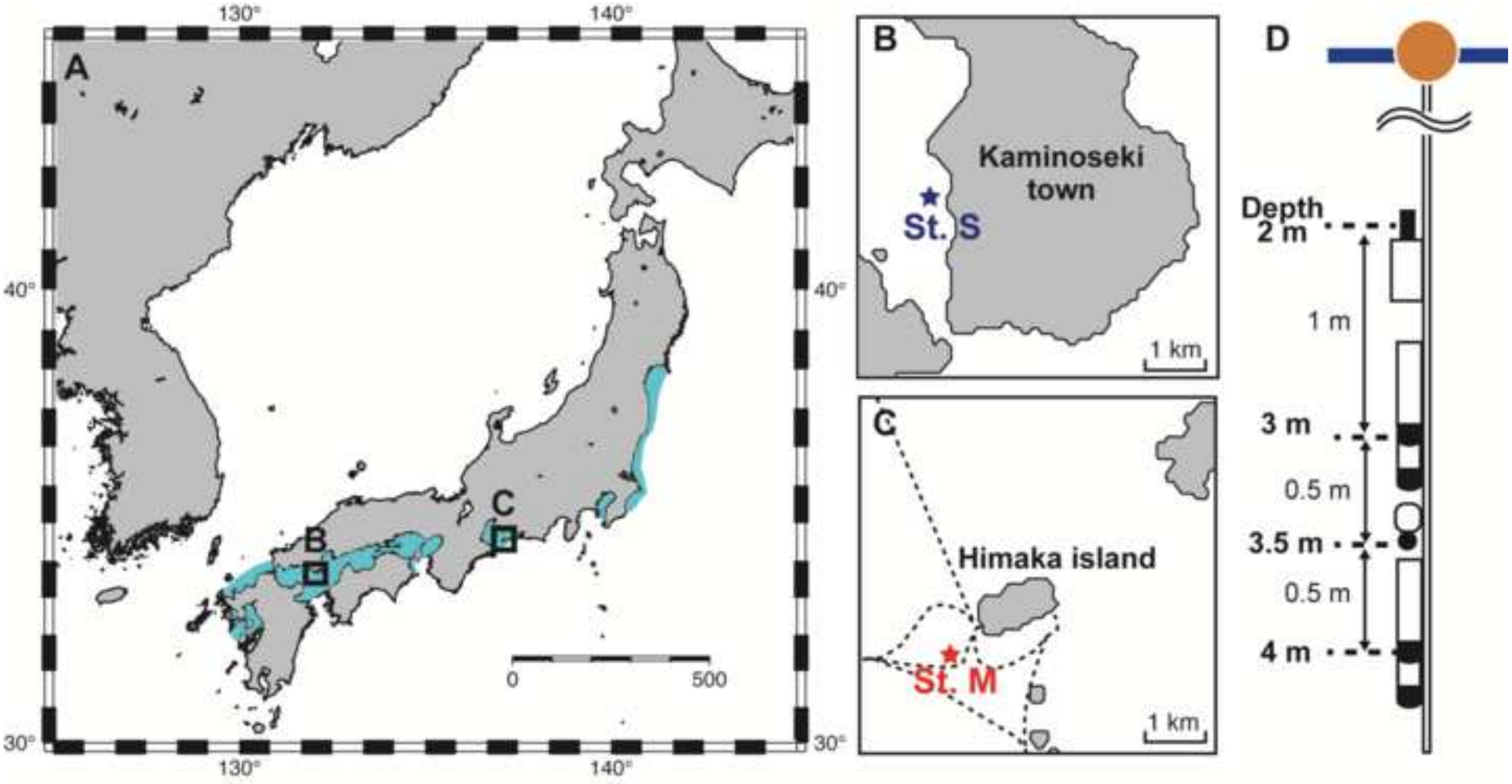

Figure_2

Click here to access/download;Figure;Fig 2_PLOSONE.tif

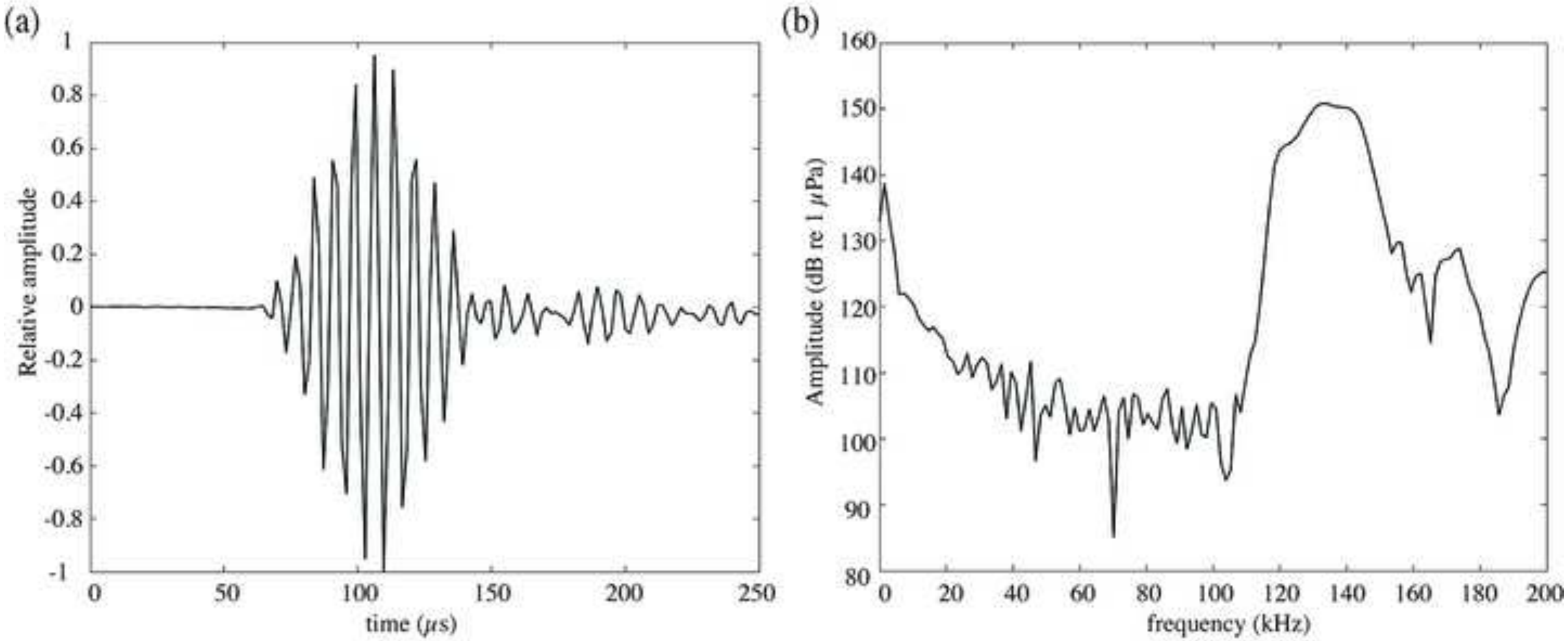

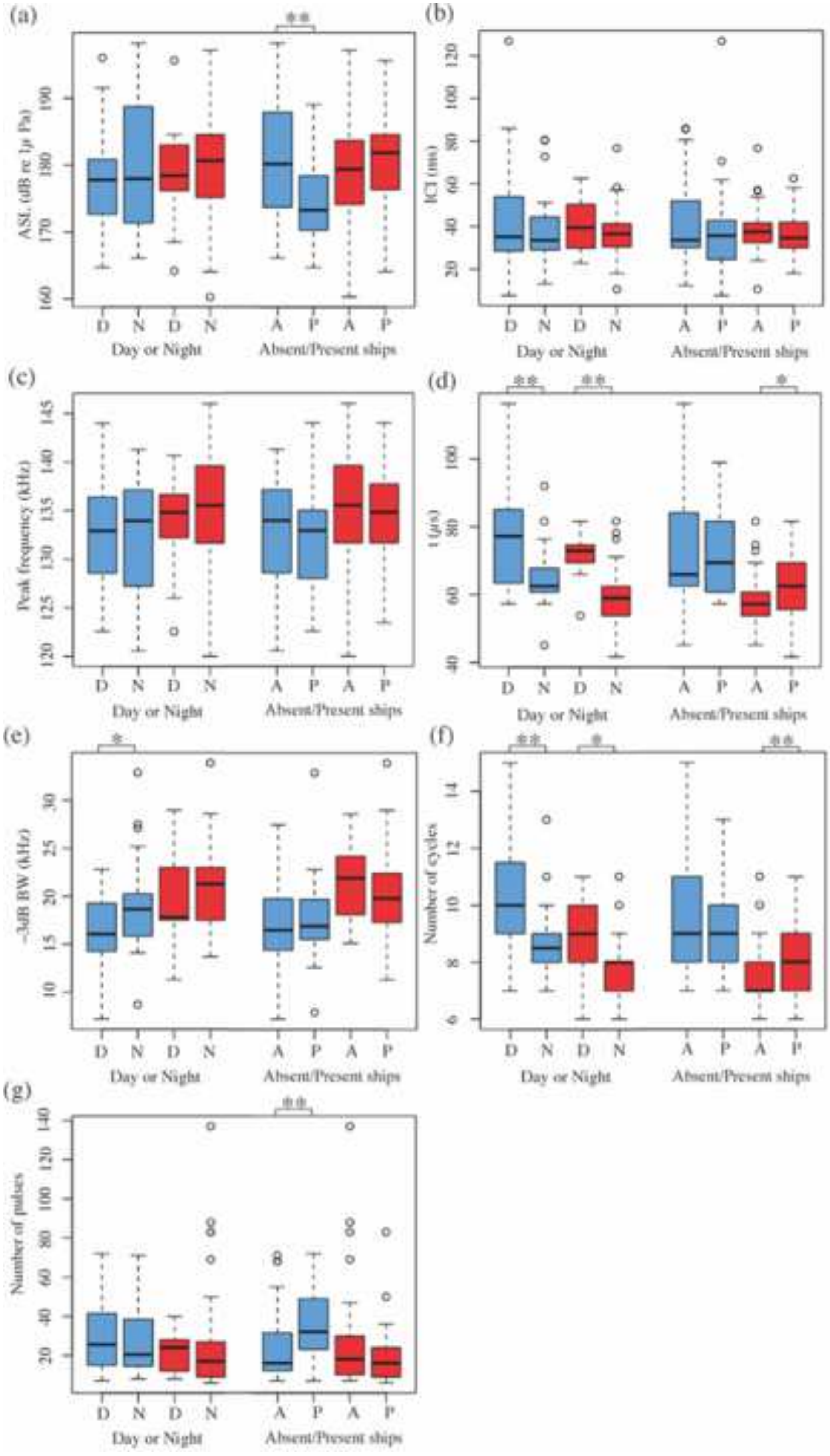
(a)
(b)
(c)
(d)
(e)
(f)
(g)
ASL (dB re 1μ Pa)
ICI (ms)
Peak frequency (kHz)
τ (μs)
-3dB BW (kHz)
Number of cycles
Number of pulses
D N D N A P A P
Day or Night
Absent/Present ships
**
*

Figure_4

Click here to access/download;Figure;Fig4_PLOSONE.tif

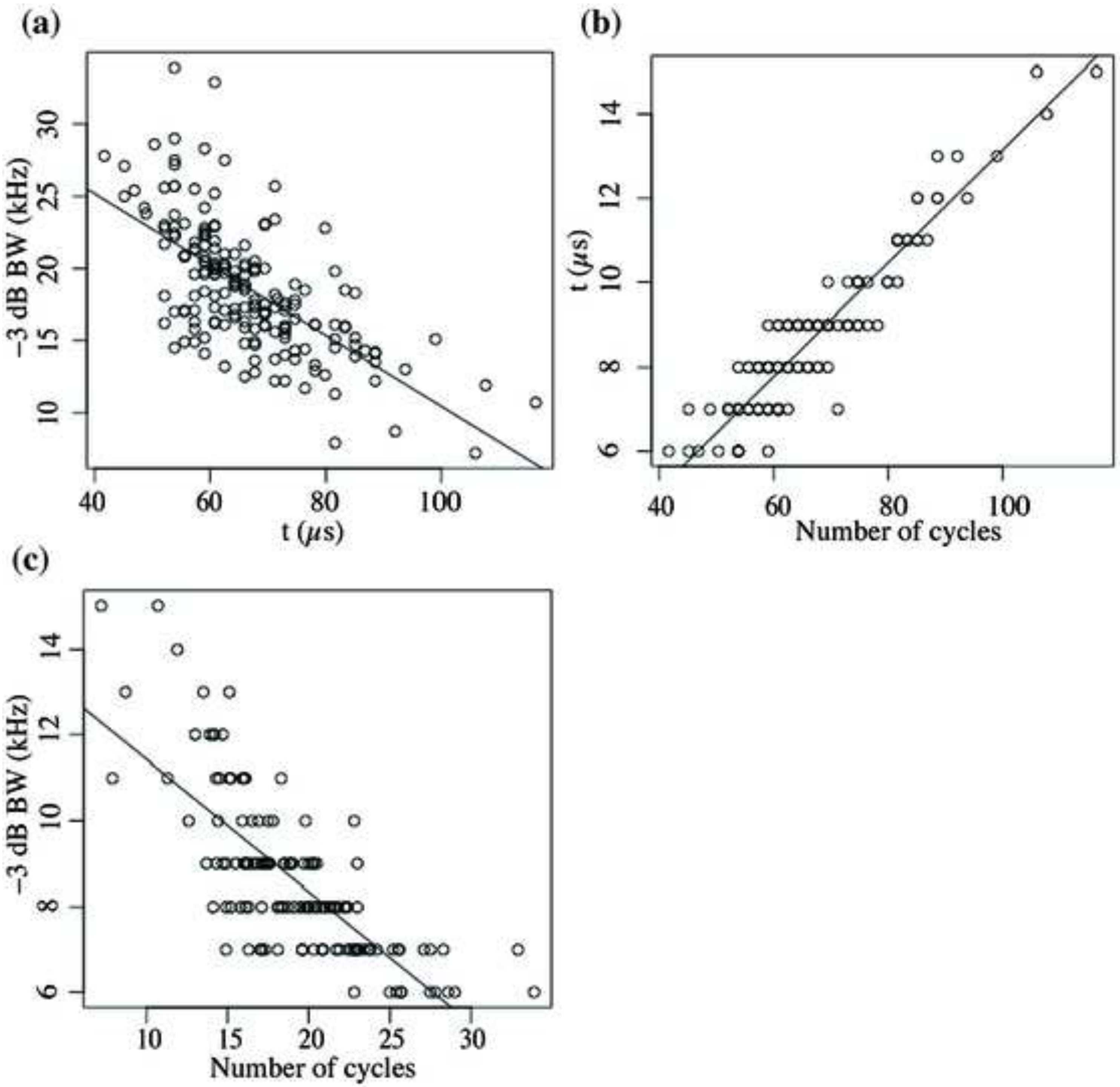

Click here to access/download;Figure;Fig5_PLOSONE.tif

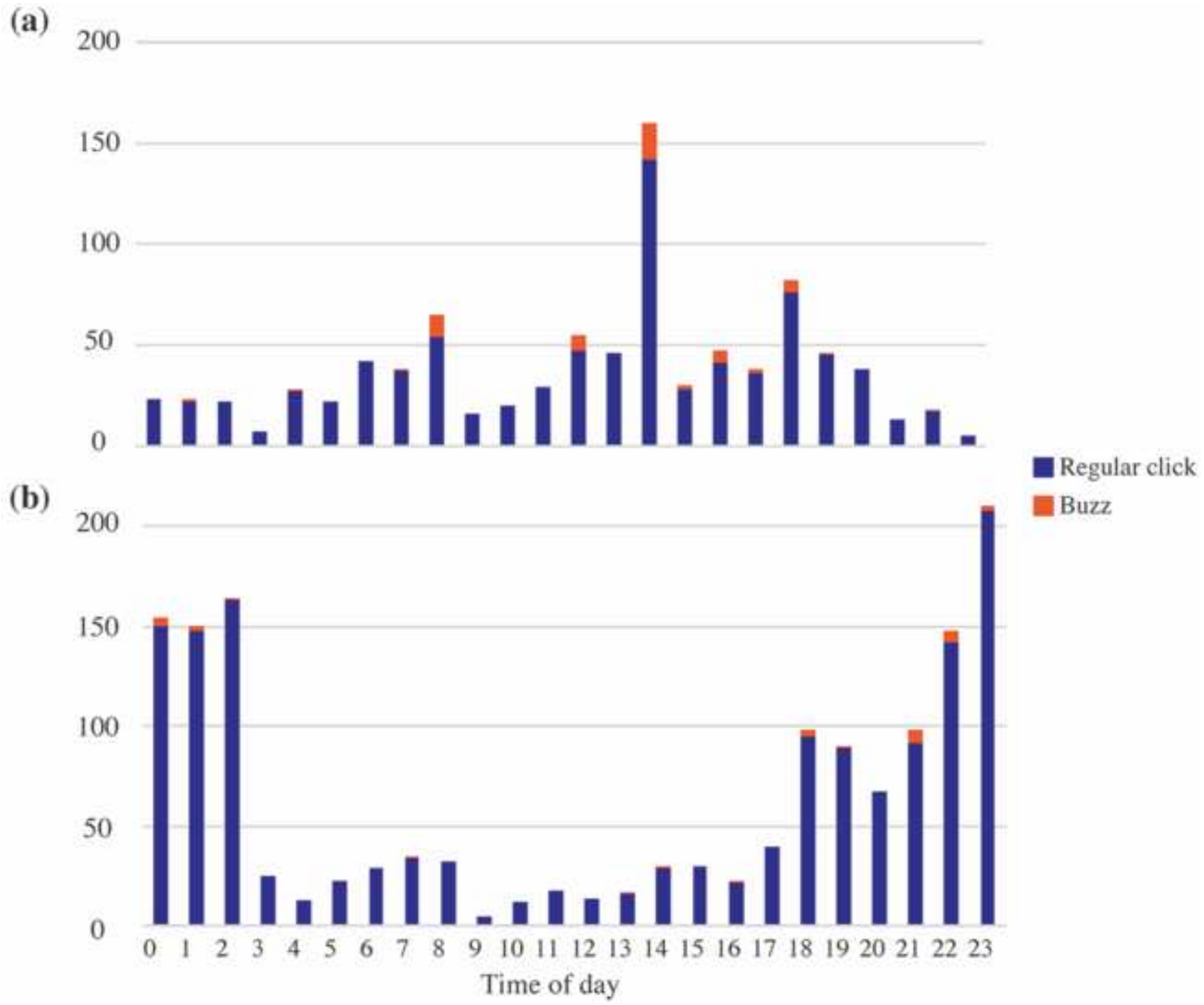

Click here to access/download
Supporting Information
Supporting_Information_PLOSONE_OgawaMayu_I.docx
