# Supplementary material for: Variations in echolocation click characteristics of finless porpoise in response to day/night and absence/presence of vessel noise
Source: PLoS One. 2023 Aug 4;18(8):e0288513. doi: 10.1371/journal.pone.0288513 (PMC10403093; doi:10.1371/journal.pone.0288513)
Supplement: Attachment — Submitted filename: response to Reviewers.docx [file pone.0288513.s005.docx]

Thank you for reading and carefully assessing our manuscript. We have made some corrections according to the journal requirements and reviewer’s comments. The journal requirements and reviewer’s comments are shown in black, while our responses are in blue, and the revised words/sentences in the manuscript are in green.

------------------------

Journal Requirements:

1. Please ensure that your manuscript meets PLOS ONE's style requirements, including those for file naming. The PLOS ONE style templates can be found at <https://journals.plos.org/plosone/s/file?id=wjVg/PLOSOne_formatting_sample_main_body.pdf> and  https://journals.plos.org/plosone/s/file?id=ba62/PLOSOne_formatting_sample_title_authors_affiliations.pdf

- Thank you for your comments. We have revised the manuscript according to the formatting guidelines of PLOS ONE.

2. We note that Figure 1 in your submission contain copyrighted images. All PLOS content is published under the Creative Commons Attribution License (CC BY 4.0), which means that the manuscript, images, and Supporting Information files will be freely available online, and any third party is permitted to access, download, copy, distribute, and use these materials in any way, even commercially, with proper attribution. For more information, see our copyright guidelines: http://journals.plos.org/plosone/s/licenses-and-copyright.

- Thank you for your comments. Figure 1 was generated by processing information provided by Geospatial information authority of Japan (Ministry of Land, Infrastructure, Transport and Tourism, https://maps.gsi.go.jp/development/ichiran.html#std) and digital national land information (Ministry of Land, Infrastructure, Transport and Tourism, https://nlftp.mlit.go.jp/ksj/gml/datalist/KsjTmplt-C23.html). The Terms of Use are compatible with the Creative Commons Attribution License 4.0 (hereinafter referred to as CC BY License), which means that the contents can be used by a third party I accordance with the CC BY license. (<https://www.gsi.go.jp/ENGLISH/page_e30286.html>, https://nlftp.mlit.go.jp/ksj/other/agreement.html; in Japanese only). Accordingly, the following sentences were added in line 115-119 and 120-122, respectively, to the caption of Table 1 “This map were modified using data set (https://cyberjapandata.gsi.go.jp/xyz/pale/{z}/{x}/{y}.png) under CC BY 4.0 and QGIS 3.30 (https://qgis.org/it/site/). Shoreline data was derived from United States National Imagery and Mapping Agency. "Vector Map Level 0 (VMAP0)." Bethesda, MD: Denver, CO: The Agency; USGS Information Services, 1997.” and These maps were modified using data set (https://nlftp.mlit.go.jp/ksj/gml/datalist/KsjTmplt-C23.html) under CC BY 4.0 and QGIS software.”

------------------------

Reviewer 1 reports:

General comments:

I read the manuscript with great interest as it contains a combination of basic understanding on echolocation of a species with red listed sub-species, and impacts of the increasing global anthropogenic threat posed by vessel noise.
The authors have recorded on- and off-axis echolocation clicks of finless porpoises and underwater sounds at two sites during approximately two months. The two sites have different number of fishing vessels in nearby harbors, and one of the sites is close to several ferry lines. In on-axis data, seven click or click train parameters are analysed and compared between the sites, between day and night, and between vessel noise absence and presence. Correlation between the on-axis click parameters are investigated. In on- and off-axis data, the total number of regular clicks and buzz-clicks are calculated per hour of day.
The manuscript is in general easy to read, although it can be improved. The data-collection and the acoustic analyses are carried out well, but I have questions on the statistical analyses of the data, and how much of the results that actually can be related to vessel (noise) impact. The results of the study are of importance for increased understanding on odontocete echolocation behaviour, impact of vessel noise, and conservation of threatened species.

- Thank you for your kind comments regarding our paper and for your suggestions. First, we increased the observation period by six months and increased the sample size to address the small sample size. Next, we made some improvements to the statistical analyses. In order to clarify the influence of vessel noises, tables were created, information was organized and disclosed. Additionally, a new GLM and GLMM was specified for statistical analysis. The explanatory variables of GLM and GLMM were absence/presence vessel noise, day/night, interaction between absence/presence vessel noise, temperature, synthetic flow velocity, and noise level. By considering the interaction between vessel noise and day/night, we were able to discuss complex changes in vessel noise and day/night, which allowed us to gain a better understanding of their relationship.

Major comments:

As I understand how the statistical analyses were carried out, there may be a confounding effect between day/night and vessel noise absence/presence. This is fundamental for the results and their interpretation.

- Thank you for your helpful comments. Based on your suggestion, we performed the statistical analysis using models, such as GLM and GLMM, to eliminate confounding factors, including environmental factors. Additionally, confounding effects between day/night and vessel noise were considered in the statistical model.

In addition to the confounding effects, there is little data supporting the conclusions on that the differences between the two sites actually is due to vessel (noise) presence. How do we know that it is not due to several other environmental factors that may vary between the sites, such as prey composition, quality and quantity? These conclusions would need a stronger basis.

- Thank you for your insightful comment. To address this issue, we increased the sample size to strengthen the results of our statistical analysis. In our previous dataset, the minimum sample size was n = 2 and the maximum sample size was n = 35, which was relatively small when divided by the combination of absence/presence vessel noise and day/night. Additionally, we acknowledged that other environmental factors could potentially contribute to the observed differences between the two sites. Therefore, several environmental factors (temperature, synthetic flow velocity, and noise level) were included in the statistical models used in this study, which was mentioned in the paragraph starting at line 241. This approach enabled us to assess the relative importance of each factor in explaining the observed differences between the sites. However, we also recognize that there may be other factors that were not considered in our statistical analyses, such as prey species or amount at the sites. To address this issue, we stated in the discussion section (line 399-405) that there were no significant differences in prey species candidates, depth, and bottom sediments between the two sites, and stated that further studies are necessary to examine the effect of these complex factors, “Furthermore, there were no differences in depth or bottom sediments at both sites. Differences in ASL at both sites are unlikely to be due to differences in feeding time or prey species. However, since the flow velocity and background noise levels between the two sites were different and the porpoises at both sites belonged to different local populations, we can not rule out the possibility that other parameters not examined in this study may have affected the behavior of the finless porpoises. Therefore, further studies are necessary to comprehensively elucidate the effect of environmental factors on finless porpoises.” Further research is needed to comprehensively understand the complex interactions between these animals and their environment.

Minor comments:

The abstract need further information for the reader to understand the work carried out.

- Thank you for your comment. We added more specific information about the aims, methods and results to help readers better understand the work performed as shown in line 20-28: “Here, we examined the effects of vessel noise and day/night on variations in echolocation clicks and click train parameters. Passive acoustic monitoring of on-axis echolocation clicks produced by free-ranging finless porpoises (Neophocaena asiaeorientalis sunameri) was performed at two sites in Japan, Seto Inland Sea and Mikawa Bay, in June–September 2021 and March–August 2022, using A-tag and SoundTrap 300HF. Generalized Linear Model and Generalized Linear Mixed Model were used to elucidate the effect of vessel noise, day/night, and surrounding environmental factors (water temperature, synthetic flow velocity, and noise level) on echolocation click and click train parameters.”

 I find the first part of the Introduction a bit unclear on what you want to present and why.

- Thank you for your feedback. Following the comments from the reviewers and based on the results obtained using a new analytical methods, we have completely revised the Introduction section. Please refer to the main text and below for more details.

I suggest additional references that may be useful (otherwise disregard them), and wonder about the links leading to web pages in Japanese.

- Thank you for your invaluable comments. Accordingly, we have replaced the links for sunrise and sunset time to one for web pages in English: “Data from timeanddata.com [56, 57] were used as a reference for sunset and sunrise information; “Yamaguchi” and “Aichi” were used as the reference points for St. S and St. M, respectively” in line 231-233. However, other references were only available in Japanese; therefore, we indicated that the webpages were “in Japanese” at the end of the reference.

The terminology of the acoustic parameters needs to be clearer (I suggest to use “click duration” instead of “t”, “clicks per train” instead of “pulses” etc), and it needs to be clarified which parameters that are click parameters and which are train parameters.

- Thank you much for observations and comments. Accordingly, we have replaced the terms “t” and “pulses” to “click duration” and “clicks” respectively, throughout the manuscript. Additionally, we ensured consistency in the use of terminologies throughout the manuscript.

I think the definition of buzz differs from what often is used, please clarify and discuss how the definition used may impact the results.

- Thank you for your comments. We changed have changed the definition of buzz to read “Five consecutive clicks with ICI < 10 ms was defined as buzz (feeding sound) [13,26]” in line 180-181. Additionally, the included the buzz ratios in the results section and discussed how the definition of buzz may affect the results: In the results, the buzz ratio of the finless porpoise in the observation sites were shown: “Moreover, the buzz ratios (percentage of total buzz to the total click train) at St. S and St. M were 8.8% (10.0% and 6.5% at day and night, respectively) and 2.9% (4.1% and 2.6% at day and night, respectively), respectively” in line 271-273, and in the discussion, it was shown that the buzz ratio was higher in the daytime at both observation sites: “In the present study, buzz ratio was higher during the day at the two sites” in line 385. These results indicated that there was no difference in day/night feeding trial rates between the sites.

I do not understand how the definition of day and night relates to the light conditions (dusk and dawn).

- Thank you for your comments. Since we already divided the data into categories such as presence/absence of vessel noise, two locations, and day/night, which is not a very large sample size even though we have increased it, we did not include dusk/night in this analysis and simply compared day and night. We added the definition of day and night as follows: “Although some diel behavioral studies on odontocetes used civil twilight times in their analyses [66], we used day and night categories (based on sunset and sunrise), due to the small sample size. The time from sunrise to sunset was defined as day, and the time from sunset to sunrise was defined as night.” in line 233-236.

The number of samples in the different categories (Site S/M, day/night and vessel noise absence/presence) need to be presented more clearly.

- Thank you for your suggestion. Accordingly, we have added information about the number of samples in different categories in Table 3.

In Results, it would be easier to follow if the results were presented in the same order as Fig. 3.

- Thank you very much for your comment. Based on your suggestion, we have changed the flow of results to match the order as Fig. 3.

As the frequency of the clicks of narrow-band high-frequency species varies extremely little, is not click duration and number of cycles per click basically two different measurements of the same thing? Are both needed?

- Thank you for your comments. As you pointed out, click duration and number of cycles per click are highly similar parameters; therefore, only click duration parameters were analysed.

Centre frequency seems to be missing in S1 Table.

- Thank you for your observation. We have added center frequency to S1 Table.

Have you considered correcting the p-value for multiple comparisons of the same data?

- Thank you for your comment. Given that the statistics analysis method was changed according to previous suggestions, GLM or GLMM *p*-value was used in the manuscript.

It is not clear why the rate of all clicks and buzzes is presented as hourly histograms and not as box plots as the on-axis click/train parameters, and there is no explanation to why all clicks and buzzes were not analysed in relation to vessel noise absence/presence. It may have been out of the scope of the study, or technical constraints, but it leaves me wondering.

- Thank you for your observation. The purpose of measuring the number of on-axis and off-axis click trains and buzzes was to examine the patterns of occurrence and the feeding activity of finless porpoise at day and night in each site. In the revised manuscript, we showed the number of click trains (exclude buzz) and buzzes in on-axis and off-axis during the day and night and buzz ratio (line 267-273): “A total of 1,257 on- and off-axis click trains (excluding buzz) were recorded at St. S (810 and 447 at day and night, respectively), while 4,104 click trains were recorded at St. M (925 and 3,179 at day and night, respectively). Additionally, 110 (81 and 29 at day and night, respectively) and 120 buzzes (38 and 82 buzzes at day and night, respectively) were recorded at St. S and St. M, respectively. Moreover, the buzz ratios (percentage of total buzz to the total click train) at St. S and St. M were 8.8% (10.0% and 6.5% at day and night, respectively) and 2.9% (4.1% and 2.6% at day and night, respectively.”

We only examined whether there was vessel noise or not before and after click events. As there was high vessel passages even during times without clicks, it was not possible to count all the ships, and thus the total number of ships at the two sites was was not reported. However, we noted the number of fishing boat in the neaby fishing ports at the site, and ferry routes and background noise levels were noted.

The sub-headings in the Discussion are helpful. Consider if the manuscript can be arranged in the same general order throughout.

- Thank you for your suggestion. We replaced the sub-headings in our manuscript as follows: in Materials and methods as “Study area and recording system”, “Echolocation click and click train analysis”, “Environmental data analysis”, and “Statistical analysis”, in Results as “Echolocation click and click train parameters,” and “GLM and GLMM model selection”, and in Discussion as “Effects of presence of vessel noise on echolocation click and click train parameters”, “Effects of day/night on echolocation click and click train parameters”, and “Comparison of echolocation click and click train parameters with previous findings”.

Overall the Discussion is supported by the Results as they are now, but need to be revised if the Results are revised (if the statistical analyses are revised).

- Thank you for your suggestion. The discussion has been revised to reflect revisions in the results.

The Discussion revolves around the energy needed for echolocation, which is relevant, but I miss further comments on the ecological implications of the findings. May prey differences between the sites explain some of the results? How does increased click duration improve echolocation during vessel noise presence?

- Thank you for your comments. Owing to the change in analysis method and results, the discussion of energy budgets was deleted in the revised manuscript. However, we included a discussion on prey species in both sites (line 385-399), “In the present study, buzz ratio was higher during the day at the two sites; additionally, there was no significant difference in the number of prey species captured in St. S and St. M. The following prey species of finless porpoise [76] were captured at St. S: whitespotted conger (*Conger myriaster*), japanease sea bass (*Lateolabrax japonicus*), east asian common octopus (*Octopus sinensis*), shrimp, and squid [77]. Prey species were captured at St. M: were japanese sardine (*Sardinops melanostictus*), dotted gizzard shad (*Konosirus punctatus*), japanese anchovy (*Engraulis japonicus*), japanese halfbeak (*Hyporhamphys sajori*), red barracuda (*Sphyraena pinguis*), japanese rockfish (*Sabastes inermis*), whitespotted conger (*Conger myriaster*), japanese sea bass (*Lateolabrax japonicus*), east asian common octopus (*Octopus sinensis*), shrimp, and squid [78,79]. Additionally, the following non-prey species of the finless porpoise were captured at both the sites: japanese jack mackerel (*Trachurus japonicus*), red tilefish (*Brachiostegus japonicus*), chubmackerel (*Scomber japonicu*), japanese spanish mackerel (*Scomberomorus niphonicus*), largehead hairtall (*Trichiurus lepturus*), righteye flounder (Pleuronectidae sp.), and bastard halibut (*Paralichthys olivaceus*), pufferfish (Tetraodonidae sp.), and crab (Brachyura). [77–79]”

And differences in prey species and feeding time did not vary among sites, indicating that finless porpoise were unlikely to have contributed to differences in ASL: “Variations in ASL at both sites may likely not be due to differences in feeding time or prey species” in line 400-401. Furthermore, the click duration was not affected by vessel noise, as indicated by the results, “For click duration, the best model was the one with the day/night (p < 0.01, S1 Table), absence/presence of vessel noise (p = 0.84, S1 Table), the interaction between day/night and absence/presence of vessel noise (p = 0.63, S1 Table), and noise level (p = 0.09, S1 Table) as the explanatory variables (AIC = 2491.7, Table 4), performed using GLMM with REML.” in line 320-323.

The Conclusions are a summary of the findings. I think it would be improved by shortening it, highlighting only the most important findings.

- Thank you for your comment. We revised the conclusion as follows to focus on the key findings of our study, while still summarizing the overall results: “n the present study, echolocation click and click train parameters were influenced by the day/night. Specifically, ASL was higher in the absence of vessel noise during the daytime at the site with low vessel traffic. Additionally, finless porpoise increased their resolution and amount of sound information by shortening the click duration, increasing -3 dB BW, and reducing ICI at night. Overall, these findings contribute to our understanding of species adaptation in response to day/night change. To accurately assess the impact of vessel noise on echolocation characteristics, it is important to consider day/night factors. However, only acoustic monitoring was performed in this study, and factors, such as the behavioral state of vocalizing individuals and the speed and size of vessels were not considered, indicating the need for further studies on the effects of these factors on echolocation.” in line 456-465.

The tables and figures are overall clear, details are commented.

- Thank you for your comments.

Please see commented manuscript for detailed comments.

- Thank you for your suggestion. We responded to the comments provided in the manuscript.

**Below is the text of Review 1 comment in the manuscript (PDF file).**

**Abstract:**

General:

As far as possible, make sure to present the measured parameters in the same order within each section of the manuscript.

- Thank you for your suggestion. We ensured consistency in the order of presenting the measured parameters throughout the manuscript.

Questions for abstract, noted when reading it before the rest of the manuscript, i.e. as a reader usually reads it. Some questions are made clear in the manuscript, but the reader needs more support here to understand what was done and what the results mean:

The reader needs basic information on the methods to be able to judge and understand the results presented in the abstract. What kind of instruments were used to measure clicks and vessel noise, and for how long were data collected? No details are needed here, just enough to help the reader interpreting the results.

- Thank you for your comments. We revised the abstract as per your suggestion as follow in line 21-28: “Passive acoustic monitoring of on-axis echolocation clicks produced by free-ranging finless porpoises (*Neophocaena asiaeorientalis sunameri*) was performed at two sites in Japan, Seto Inland Sea and Mikawa Bay, in June–September 2021 and March–August 2022, using A-tag and SoundTrap 300HF. Generalized Linear Model and Generalized Linear Mixed Model were used to elucidate the effect of vessel noise, day/night, and surrounding environmental factors (water temperature, synthetic flow velocity, and noise level) on echolocation click and click train parameters.”

It is confusing about what was tested, and why. The first comparison is diel (what is that? day-night only, or four phases?) for the entire dataset, then within the quieter site (before-after vessel, unclear what happened to the diel phases), then within the noisier site (not explaining but probably before-after vessel?, and again, what happened to the diel phases?), then between the sites (what happened to diel phases and before-after vessel here?). At the end conclusions are given in relation to vessel noise, but it is very unclear how the conclusions were reached. How come the differences acoustic behaviour is simply not due to differences in other environmental parameters between the sites?

- Thank you for your comments. Based on the suggestion of Reviewer 1, the statistical analysis method was revised. Accordingly, the manuscript was revised to focuse on the effects of absence/presence of vessel noise and day/night on echolocation parameters of finless porpoises, the differences in these parameters between the two study sites, and the confounding effect. The absence/presence of vessel noise, day/night, water temperature, synthetic flow velocity, and noise level were used as explanatory variables, and the click and click train parameters were used as response variables in GLM and GLMM. Reanalysis revealed that day/night significantly affected echolocation characteristics. In contrast, other environmental factors had a small effects, which were not significant. The following text was added in the abstract as follow in line 21-28 and 32-34: “Passive acoustic monitoring of on-axis echolocation clicks produced by free-ranging finless porpoises (*Neophocaena asiaeorientalis sunameri*) was performed at two sites in Japan, Seto Inland Sea and Mikawa Bay, in June–September 2021 and March–August 2022, using A-tag and SoundTrap 300HF. Generalized Linear Model and Generalized Linear Mixed Model were used to elucidate the effect of vessel noise, day/night, and surrounding environmental factors (water temperature, synthetic flow velocity, and noise level) on echolocation click and click train parameters.” and “Overall, these results suggest that echolocation clicks by finless porpoise were likely to fluctuate to adapt with surrounding complex environmental conditions, especially day/night.”.

L23: What does this mean? Was the click duration shorter at night regardless of if it was at night or day??

- Thank you for your comments. The click duration was shorter at night regardless of the absence/presence vessel noise. The indicated this in the abstract (line 29-31): “Particularly, -3 dB bandwidth was wider, click duration was shorter, and inter-click intervals in a train were shorter at night”.

L23: Better to use the term "duration"

- Thank you for your comments. The term “duration” was used in the manuscript instead of “length”.

L24: Shall be "and"?

- Thank you for your observation. This sentence was deleted from the abstract, as it was unclear. Moreover, the information was not important in the abstract section.

L24: Difficult to understand what this means without any explanation. This is not mentioned elsewhere in the abstract, making it a bit confusing about what acoustic parameters that were used in which comparisons and why.

- Thank you for your comments. Owing to the change in analysis method, this sentence was deleted, and the ratio of on- and off-axis click trains and buzzes was not key aim of the study, so we deleted this sentence.

L25: Replace by "between"

- Thank you for your suggestion. Owing to the change in analysis method, this sentence was deleted.

L25: Can be deleted, does not add anything to what already has been said in the first part of the sentence.

- Thank you for your suggestion. We deleted this sentence.

L26: Be consistent in what term is used, replace by "site".

- Thank you for your comments. We replaced from “area” and “location” with “site” in though manuscripts.

L27: More straightforward to call it "clicks per train", instead of introducing "pulse" as an alternative term to "click".

- Thank you for your suggestion. We changed to “clicks per train” or “clicks in a train” in though manuscripts.

L27: Delete

L28: Replace by "compared to"

- We deleted this sentence because the statistical analysis method was changed, based on the isstruction of the reviewer.

L28: Site

- We changed to “site” throughout this manuscript.

L29: duration

- We changed to “duration” throughout this manuscript.

L29: Delete

- Owing to the fact that the statistical analysis method was changed, this sentence was deleted.

L30: As this is contrasting to the results at the first site, and is given first in the sentence about the other site, this should also be presented first in this sentence. OR this contrasting result should be lifted to an own sentence explaining the opposite results from the two sites, and the other site-specific significant results explained in another sentence.

- Thank you for your comments. This sentence has been deleted, as it as affected by the change in statistical method.

L30: duration

- Thank you for your observation, it has been changed to “duration” throughout the manuscript.

L31: clicks per train

- This sentence was deleted because of changes in the results. However, “number of pulses” was changed to “number of clicks per train” in the manuscript.

L33: How can you separate differences if the click parameters between the sites are due to differences in vessel noise or other environmental parameters? If you cannot, the "response differences" cannot be attributed to the different levels of vessel noise between the sites.

- Thank you for your comment. We changed the analysis method to GLM and GLMM so that other environmental parameters, such as water temperature, can be taken into consideration. The new analysis revealed that day/night significantly affected click characteristics.

L35: How come this is concluded to be the primarily regulation when most investigated parameters vary within or between sites?

- Thank you for your comment. This sentence has been deleted due to a change in the analysis method, as previously suggested by Reviewer 1.

L35: duration

- We changed the term to “duration” throughout the manuscript.

**Introduction**

L38: I'm sorry, but I do not understand what you want to say with this part of the introduction. You say that it is challenging to study echolocation clicks of free ranging animals and give a 15 year old reference on technological suggestions on how to study this (#2), followed by listing 11 references (#6-16) where is has been done. Then you say that acoustic studies mainly are carried out in conjunction by visual studies during daytime (listing references #7-15), followed by saying that diel acoustic behaviour in odontocetes has been extensively studied by using PAM and tags (listing references #17, 23-29). If acoustic observations have been combined with visual or not usually varies with the research question, some requires visual observations and others don't. As it is now, it rather seems to be a description of how much that is read about this topic, than presenting a thought-through context to your study. In those cases there are many available references to support what you present, chose only the most relevant ones (max ~4) instead of listing all available. Other possibly useful references on the topic are (depending on what you want to present):

Koblitz, J.C., Wahlberg, M., Stilz, P., Madsen, P.T., Beedholm, K., Schnitzler, H.-U., 2012. Asymmetry and dynamics of a narrow sonar beam in an echolocating harbor porpoise. J. Acoust. Soc. Am. 131, 2315–2324. https://doi.org/10.1121/1.3683254

Kyhn, L.A., Tougaard, J., Beedholm, K., Jensen, F.H., Ashe, E., Williams, R., Madsen, P.T., 2013. Clicking in a killer whale habitat: narrow-band, high-frequency biosonar clicks of harbour porpoise (*Phocoena phocoena*) and Dall’s porpoise (*Phocoenoides dalli*). PloS One 8, e63763. https://doi.org/10.1371/journal.pone.0063763

Wisniewska, D.M., Johnson, M., Beedholm, K., Wahlberg, M., Madsen, P.T., 2012. Acoustic gaze adjustments during active target selection in echolocating porpoises. J. Exp. Biol. 215, 4358–4373. https://doi.org/10.1242/jeb.074013

- Thank you for your helpful comments. Based on your comments, the introduction was edited to clearly indicate the aim of the study. First, we described how odontocetes are affected by the vessel noise as follow in line 42-48: “Over the years, several studies have been performed to elucidate the impact of vessel noise on echolocation clicks. For instance, atlantic bottlenose dolphins (*Tursiops truncatus*) and harbor porpoises *(Phocoena phocoena)* decreased emitting buzz rate, feeding trial sound, and echolocation click [13,14], in response to the presence of vessels, indicating a decrease in their feeding activity [15,16]. Additionally, Lahille’s bottlenose dolphins (*T. truncatus gephyreus*) emitted significantly fewer echolocation clicks in the presence of vessels in ~ 250-m radius [17]..” Then, we highlighted the issue regarding the effect of vessel noise on odontocetes in line 48-52: “Moreover, melon-headed whales (*Peponocephala electra*) increased echolocation click source level in correlation with the level of ambient sound pressure [18]. However, previous studies were mainly focused on the effects of vessel noise on the echolocation click train emitting rate, with limited studies on variations in echolocation click and click train parameters [18].” Additionally, we described the effect of day/night on click and click train parameters in odontocetes in line 58-68, “Previous findings suggest that echolocation click and click train parameters are affected by the surrounding environment [21], and diel changes in echolocation click characteristics have been observed in some species. For instance, melon-headed whales exhibit higher center frequencies at night owing to increased feeding activity [18], and harbor porpoises emitted high proportion of click trains with short inter-click intervals (ICIs) to forage or investigate objects at close range [22]. Additionally, the diel behavior of harbor porpoises likely depends on changes in the diel behavior of their prey [23–26]. However, studies on the variations in echolocation click characteristics during the day/night have not been conducted in several species, and it is unclear if diel variations in echolocation click are associated with vessel noise. Therefore, it is necessary to examine variations in echolocation click during the day/night and in the absence/presence of vessel noise.” Lastly, we justified why finless porpoise was selected in this study in line 78-84: “Recently, some studies have examined the impact of vessel noise on Yangtze finless porpoise. For example, increase in vessel noise have been shown to affect Yangtze finless porpoise distribution [36,37], cortisol levels [38], and activity [39]. Additionally, Yangtze finless porpoise emit more buzz at night for foraging [35,40], finless porpoises avoid passing boats by diving [41]. However, relatively few studies have investigated changes in echolocation click and click train characteristics for small odontocetes, and no study has been conducted on finless porpoises.” Additionally, relevant references were cited to support the issues introduced in the introduction section. Although the references you provided were informative, we did not cite Koblitz et al. (2012) and Wisniewska et al. (2012) because we felt they were not entirely consistent with what we wanted to explain in the Introduction. Regarding Kyhn et al (2012), it was cited as Ref 84 in the discussion.

L39: Delete comma

- Thank you for your comment. We have deleted the comma.

L 40: I don't understand what you want to say by this clause. The study is on free-ranging animals, which indeed is challenging, but here it is just stated that it is challenging in general. Further, since then

- Thank you for your comments. We intended to state that there have been very few impact assessment studies on the effect of vessel noise on echolocation clicks. The sentences have been revised accordingly in line 48-50: “Moreover, melon-headed whales (*Peponocephala electra*) increased echolocation click source level in correlation with the level of ambient sound pressure [18].”

L51: Two other PAM studies investigating the impact on environmental factors and diel patterns of harbour porpoises that are worth considering here are:

Clausen, K.T., Teilmann, J., Wisniewska, D.M., Balle, J.D., Delefosse, M., van Beest, F.M., 2021. Echolocation activity of harbour porpoises, Phocoena phocoena, shows seasonal artificial reef attraction despite elevated noise levels close to oil and gas platforms. Ecol. Solut. Evid. 2, e12055. https://doi.org/10.1002/2688-8319.12055

Zein, B., Woelfing, B., Dähne, M., Schaffeld, T., Ludwig, S., Rye, J.H., Baltzer, J., Ruser, A., Siebert, U., 2019. Time and tide: Seasonal, diel and tidal rhythms in Wadden Sea Harbour porpoises (Phocoena phocoena). PLOS ONE 14, e0213348. <https://doi.org/10.1371/journal.pone.0213348>

- Thank you for your suggestion. The studies have been cited and the references have been added in the revised manuscript in line 63-64: “Additionally, the diel behavior of harbor porpoises likely depends on changes in the diel behavior of their prey [23–26].” Ref 22 is for Clause et al. (2021) and 25 is for Zein et al. (2019).

L61: The sentence gives a statement on all aquatic species, but 3 of the references are on odontocetes and 1 is on fish. If you want to give a broad statement, your references also need to support that. Examples of a multi-species and a fish reference (there are many more on fish available):

Duarte, C.M., Chapuis, L., Collin, S.P., Costa, D.P., Devassy, R.P., Eguiluz, V.M., Erbe, C., Gordon, T.A.C., Halpern, B.S., Harding, H.R., Havlik, M.N., Meekan, M., Merchant, N.D., Miksis-Olds, J.L., Parsons, M., Predragovic, M., Radford, A.N., Radford, C.A., Simpson, S.D., Slabbekoorn, H., Staaterman, E., Van Opzeeland, I.C., Winderen, J., Zhang, X., Juanes, F., 2021. The soundscape of the Anthropocene ocean. Science 371, eaba4658. https://doi.org/10.1126/science.aba4658

Popper, A. n., Hastings, M. c., 2009. The effects of anthropogenic sources of sound on fishes. J. Fish Biol. 75, 455–489. <https://doi.org/10.1111/j.1095-8649.2009.02319.x>

- Thank you for your suggestion. The studies have been cited and the references added in the revised manuscript in line 40-42: “Therefore, the impact of vessel noise on behavior and vocalization in aquatic species [6–12], including small odontocetes [6–9], has attracted increasing attention.” Ref 9 is for Duarte et al. (2021) and 11 is for Popper et al. (2009). In addition, a paper related to invertebrates was also cited as Ref 12.

L62: This has turned out a bit strange, referring to a sperm whale (#37) and a beaked whale paper (#38) for definition of buzzes in Tursiops and harbour porpoises. All these species have buzzes, but they are very different. I would recommend to instead start by a sentence saying that many odontocete species produce buzzes, indicating foraging behaviour (also suitable to add Verfuss et al. 2009, J Exp Biol), and in the following sentences give examples of observed changes in buzz rate due to increased noise levels in various species).

- Thank you for your comments and suggestions. Based on your suggestion, we also cited studies on harbor porpoise, a species closely related to finless porpoise, the target species of this study (line 43-46), “For instance, Atlantic bottlenose dolphins (*Tursiops truncatus*) and harbor porpoises (*Phocoena phocoena*) decreased emitting buzz rate, feeding trial sound and one of the echolocation click [13,14], in response to the presence of vessels, indicating a decrease in their feeding activity [15,16].” Ref 13 is for Verfuß et al. (2009) and 14 is for Akamatsu et al. (2010).

L68: This reference is about Ganges river dolphin, # 43 is about Lahille's dolphin

- Thank you for your observation. We have corrected the citation, “Additionally, Lahille’s bottlenose dolphins (*T. truncatus gephyreus*) emitted significantly fewer echolocation clicks in the presence of vessels in ~ 250-m radius [17].” in line 46-48. Ref 17 is for Pellegrini et al. (2021).

L71: Mean "vessel noise"? Ship strikes is another type of problem caused by vessels.

L72: How can a single-species study conclude that the impact varies among species? Is some other reference meant here?

- Thank you for your comments. The introduction has been significantly revised, resulting in the deletion of this sentence in L38 of previous version (above).

L80: Rephrase as: "distribution and cortisol levels of Yangtze finless porpoise [46-48]", or "distribution [46,47] and cortisol levels of Yangtze finless porpoise [48]".

- Thank you for your comments. The sentence has been revised to clearly indicate that Yangtze finless porpoise is the species of interest in line 79-80: “For example, increase in vessel noise have been shown to affect Yangtze finless porpoise distribution [36,37], cortisol levels [38], and activity [39].”

L84: A bit strange to start a paragraph by "Therefore", delete.

- Thank you for your insightful comment. We have deleted “Therefore”.

L86: What is meant by this term? Only the physical properties of the emitted sounds, or also the behavioural aspects of the animals' sound production? I have understood that you also want to study the acoustic behaviour, but cannot see that this is covered by the term used.

- Thank you for your observation. In line with the objectives of this study (to examine the click and click train parameters), we replaced “sound characteristics” with “click and click train parameters”.

**Materials and Methods**

L101: boats

- Thank you for this correction. It has been effected in line 110.

L103: As far as I can see, the linked pages are in Japanese only, making it difficult for many readers to understand them. Also, should they not be given as references following PlosOne's guidelines? <https://journals.plos.org/plosone/s/submission-guidelines#loc-references>

- Thank you for your comments. This information is only available in Japanese; therefore, we added "in Japanese" at the end of the reference [48] and [49]. Additionally, we followed the PLOS ONE guideline for citation and formatting of the reference list.

L109: From where is this information? A reference is needed. Add a north arrow to the map?

- Thank you for your comments. We added the reference in the figure citation, as follows: “(a) Distribution of finless porpoise in the Japanese coastal areas (blue) [46]” in line 114-115. Additionally, the north arrow has been added in Fig. 1. Reference [46] is for Yoshida et al. (2002).

L110: I think an explanation like "The locations of the arrays is shown by the stars." should be added to (B, C). Then (D) should read something like "Configuration of the arrays."

- Thank you for your comments. We revised the figure caption, as follows: “The locations of the arrays are depicted by circles, and the dotted lines denote the ferry routes. (d) Configuration of the arrays. From the top, the instruments (black in the figure) were placed with their sensors at 2 m (INFINITY-EM), 3 m (A-tag), 3.5 m (SoundTrap), and 4 m (A-tag) from the water surface (blue line).” in line 122-125.

L111: routes

- We changed the term from “route” to “routes” in line 123.

L111: Replace by "instruments"

- Thank you for your suggestion. We replaced “sensors” with “instruments” in line 123.

L112: Add "with their sensors"

- Thank you for your suggestion. We added “with their sensors” in line 124.

L118: As the current meter does not record acoustic data, this needs to be rephrased.

- Thank you for your comment. We have indicated that only “Acoustic and environmental data” were recorded (line 130).

L120: Replace by "The instruments were assembled on a vertical array" (also solves the problem with the current meter not being an acoustic instrument).

- Thank you for your comment. We revised “An acoustic array was assembled in a vertical position” to “The instruments were assembled on a vertical array” (line 132-133).

L122: Replace by "from". It is explained in the following sentence that buoys were used.

- Thank you for your comment. We changed “with buoys attached at the” to “from” as shown in line 134.

L123: Delete, as the current meter is not an acoustic instrument. You can say e.g. "monitoring arrays" if you like.

- Thank you for your comment. We have added “monitoring arrays” in line 134.

L123: Replace by "attached to surface buoys". Possibly also add to the sentence that the arrays were anchored?

- Thank you for your comment. We added “attached to surface buoys, which were anchored” (line 134-135).

L125: See above

- Thank you for your help. We deleted “acoustic” as shown in line 136: “Data were obtained by multiple continuous recordings for ~ 8 d”.

L129: Reaching this, I miss an explanation (with references) in the Introduction to that the species (like many other odontocetes) emit fast-repetition series of clicks called 'click trains'.

- Thank you for your comments. We inserted “Both finless porpoise and Yagntze finless porpoise do not emit whistle, but emit narrow-band high frequency echolocation clicks [21,29–31]. Additionally, Yangtze finless porpoise emits an average of one click train every 5–6 seconds [32–35].” in the introduction in line 74-77.

L130: Replace by "of consecutive clicks"? Already said in the sentence that the trains were detected this way.

- Thank you for your comments. We added “of the consecutive clicks” in line 143.

L147: Replace by "as"

- Thank you for pointing this out. We added “as” in line 159-160, as follow: “Synthetic flow velocities were as one of the environment factors”.

L149: Replace by "recorded during"

- Thank you for your comment. We deleted this sentence because this data were not included in the analysis due to missing environemental data.

L166: Replace by "the arrival time"?

- Thank you for your comment. We added “the click arrival time” as shown in line 184.

L171: The amplitude of longer porpoise click train series are often "wave-shaped" (going up and down several times) due to the head movements resulting from the fluke beats. How does this relate this criterion? Was one tail beat considered as one click train?

- Thank you for pointing this out. We revised “(3) the series of click trains had amplitudes that first increased and then decreased” to “the amplitude of the series of click trains should first increase and then decrease (*sensu* [64])” as shown in 188-189. A click train (without noise) usually starts with small amplitude, which increases and then finally decreases again.

L172: This is not a criterion by itself, must be linked to something more.

- Thank you for your comments. We have removed this criterion in the revised manuscript and replaced it with other information as shown in line 192-193: “Clicks with the highest amplitude in the series of click trains were analyzed.”

L173: More of interest: were there always multi-path clicks associated to the on-axis click? If not, add ", when present" or similar at the end of the criterion.

- Thank you for your suggestion. We added “when present” as shown in line 191.

L181: Reading the explanations of the parameters below, it becomes clear that the parameters listed here are a mix of click and train parameters. This needs to be made clear. Make sure the terms used reflects this.

The click parameters are (1 measurement per click):ASL, click duration, number of cycles

The train parameters are (1 measurement per train):ICI, peak frequency, center frequency, bandwidth, number of clicks per train

Or was there only 1 on-axis click per train, and thereby also only 1 measurement of each click parameter per train?

- Thank you for your suggestion. We revised this section to clearly state the click parameters and the click train parameters, as shown in line 199-203: “Click and click train parameters for on-axis echolocation click of finless porpoises were calculated using MATLAB. Click parameters included the apparent source level (ASL) in dB re 1 μPa, peak frequency and center frequency in kHz, -3 dB bandwidth (BW) in kHz, click duration in μs, as well as click train parameters such as ICI in milliseconds (ms) and the number of clicks per train”.

L181: Delete or maybe replace by "sound". Besides ASL, all parameters are received and not source parameters (even though it is assumed that the difference is minor for most of them, it is likely major for the number of clicks per train).

- Thank you for your comments. We replaced this with “click parameters” and “click train parameters,” as shown in line 200 and 202.

L183: click duration?

- Thank you for your suggestion. We used “click duration” accordingly in line 201-202.

L183: Isn't this just another way of expressing t, if the frequency is held constant? Are both t and number of cycles needed?

- Thank you for your suggestion. Since number of the cycles and click duration (t) are the same, only click duration (t) was used in this manuscript. Additionally, the description was changed from t to “click duration.”

L183: number of clicks per train?

- Thank you for your suggestion. We replaced “number of pulses” to “number of clicks per train” as shown in line 202-203.

L192: Long sentence. Replace by: “, with the exception of July 6-12 when no data was obtained from St. S. Instead, data”

- Thank you for your comment. We deleted this sentence because this data were not included in the analysis due to missing environemental data.

L195: Same as previous links

- This information is only available in Japanese; therefore, we added "in Japanese " at the end of reference [63] and [64] in the reference list. We followed the PLOS ONE guidelines for citation.

L196: Unclear statement, rephrase.

- We changed the sentence as follow: “Salinity data at St. S were obtained from the Hirae Iwakuni fixed-line survey conducted by the Inland Sea Research Department of the Yamaguchi Prefectural Fisheries Research Center at 33°50’12″N, 132°02’04″E (~ 7.7 km from St. S) once a month during the same period, using the Yamaguchi Prefecture submarine and fisheries research vessel “Seto” [64]” in line 210-214.

L199: To facilitate for the reader to judge this, you could calculate the distance between the array and this station and add that (", located approximately XX [unit] from the monitoring array").

- Thank you for your suggestion. We revised the sentence as follows: “Salinity data at St. S were obtained from the Hirae Iwakuni fixed-line survey conducted by the Inland Sea Research Department of the Yamaguchi Prefectural Fisheries Research Center at 33°50’12″N, 132°02’04″E (~ 7.7 km from St. S) once a month during the same period, using the Yamaguchi Prefecture submarine and fisheries research vessel “Seto” [64]. Salinity data were measured at St. M at a 3.5 m depth, using the No. 2 Automatic Oceanographic Observation Buoy in Mikawa Bay (34°44’42″N, 137°04’19″E; ~ 9 km from St. M), which is owned by the Aichi Fisheries Research Institute [65].” in lines 210–217.

L200: Same as previous links

- This information is only available in Japanese; therefore, we added "in Japanese" at the end of references [64] and [65] in the reference list. We followed the PLOS ONE guidelines for citation.

L201: Assuming the max amplitude only was measured at one click in the train, I recommend rephrasing the sentence as:

"The ICI was calculated as the average interval between the clicks before and after that with the maximum amplitude in each train, i.e. as the average of the two ICIs before and after the click with the maximum amplitude."

L202: "as the" missing here?

L202: Delete, ICI is a time interval (not a sound interval).

L202: Replace by "between"

- Thank you for your comments. We truly appreciate your detailed and kind review. We have revised the sentence to read “ICI was calculated as the average interval between two ICIs before and after the click with the maximum amplitude” in line 2324–225.

L206: click duration?

- We changed “t” to “click duration”.

L208: Replace by e.g. "finally, the number of clicks per train was measured".

- We have revised the sentence to “Finally, the number of clicks per train was measured” in line 225-226.

L211: "click trains"?

- We revised it to “click trains” here.

L211: Replace by "at each site".

- We replaced with “at each site” in line 231.

L212: Same as for previous links.

Also, how does this information relate to the hours used for definition of day and night below?

- Thank you for your comments. We added the link to the English version here and added the definition of day/night “Data from timeanddata.com [56, 57] were used as a reference for sunset and sunrise information; “Yamaguchi” and “Aichi” were used as the reference points for St. S and St. M, respectively.” as shown in line 231-233.

L216: Better called "vessel noise"? Later sometimes called "vessel traffic", "absent/present ships" etc, make sure to chose one appropriate term and use that consistently.

- Thank you for your suggestion. We used “vessel noise” throughout the manuscript.

L217: Must be "click trains"? Single clicks were rejected.

L218: Same as above, "click train"?

L221: Replace by "and click train"?

- Thank you for your comments. We changed these to “click trains.”

L223 : Replace by "used to analyze possible correlations".

- Thank you for your suggestion. We deleted this sentence due to the change in the analysis method, as suggested in previous comments.

L224: Replace by "possible correlations between all click and click train parameters, and"

- Thank you for your suggestion. We deleted this sentence due to the change in the analysis method. The relationship between environmental parameters (water temperature, synthetic flow velocity, noise level) and click or click train parameters was analyzed using GLM and GLMM.

L226: Replace by "investigated" or "analyzed"

- Thank you for your suggestion. We removed this sentence because we revised the analysis method, based on the previous comments.

L227: The ratio and not the rate was calculated, see comment on Results

- Thank you for pointing this out. We changed “rate” to “ratio” in line 181.

L228: Or click trains?

- We changed “click” to “click trains.”

L229: Unclear: above is an explanation of how "the ICI" was calculated, is "the ICI" the same as ""average ICI"? If the same parameter is meant, make sure to give it a clear term, and use that term consistently. "Click trains with an average ICI <10 ms" sounds like the average ICI of *all* ICIs in the train is meant. Also, and very important, a common definition of a buzz in studies using PODs (by Chelonia Ltd) is a train or a time unit with five consecutive clicks with ICI <10 ms (see e.g. Zein et al. 2019 PlosOne and references therein). As buzzes most often occur at the end of a train, and you define a buzz as a train with ICI <10 ms around the click of the max amplitude, it is likely that you get a much lower proportion of ICIs than in studies using the other definition, as the max amplitude is less likely to occur at close distance to the target just before a capture attempt.

- Thank you for your insightful comment. We changed the definition of buzz and added the following sentence in line 180-181, “Five consecutive clicks with ICI < 10 ms was defined as buzz (feeding sound) [13,26]”, reference [26] is for Zein et al. (2019).

L230 : What do you mean by this? Reading the results, it was done per site and separately for night and day, and not pooled across the entire dataset.

- Thank you for your comments. We revised this sentence as follows: “Additionally, click trains and buzzes were categorized as day/night [56,57], and their hourly average was calculated” in line 182-183.

L230: buzzes

- We changed “buzz” to “buzzes” in line 181.

L231: How do these hours relate to the sunrise and sunset information given above?

- Thank you for your comment. We used the same definitions for day/night for the classification of click and click train parameters, which was clarified in line 182-183 “Additionally, click trains and buzzes were categorized as day/night [56,57], and their hourly average was calculated.”.

**Result**

L233: It is not possible to fully evaluate the results of the click parameters due to that:

Sample sizes are not presented.

- Thank you for your comment. We added the sample size in Table 3.

It is unclear why the data first were divided and compared between sites, and then compared again within site between day/night (pooling vessel absent/present) and between vessel absent/present (pooling day/night). These partially pooled comparisons are only OK to do if it is assumed that there is no interaction between day/night vs vessel absent/present and the sampling is balanced. For example: click duration was found to be longer during the day than during the night at both sites (Fig 3d). Then if most samples with vessels present are sampled during the day, and most samples with vessels absent are sampled during the night, it will erroneously seem like vessel presence causes longer clicks, while in reality it was an effect of day.

- Thank you for your comments and suggestions. This point was also raised in the major comments by the same reviewer. Based on these comments, we analyzed the data using GLM and GLMM to account for the confounding effects of absence/presence of vessel noise and day/night. GLM was used when click and click train parameters were not significantly different between the sites, and GLMM was used when these parameters were significantly different. Since there were changes in the result due to due to the change in analysis method, the discussion was based on the revised results (see Results and Discussion parts in the revised manuscript).

L234: I am lost here, do you mean clicks (i.e. individual pulses) or click trains? It is very important that the sampling unit is clear. If you have used data for more than one click per train, these samples are not independent and should be grouped within train before you do any comparisons.

- Thank you for your comment. We meant click trains, i.e. changed this to “click trains” as in line 261. We calculated the click parameter for only one of the click trains, as shown in line 192-193: “Clicks with the highest amplitude in the series of click trains were analyzed”. We added the sample size in Table 3.

L235 The number of clicks is actually not shown in Fig 2, but it would be helpful of this also was included (if these numbers indeed are the number of samples).

- Thank you for your observation and suggestion. Click, click train, and environmental parameters have been described in Table 2. We believe you are referring to Table 2 and not Figure 2. Therefore, we have added the sample sizes to Table 2.

L235: Maybe better presented as a table showing the number of clicks per site, day/night, and vessel absence/presence? See also comment sample sizes on Fig 3.

- Thank you for your comment. For ease of understanding, we added the sample sizes for the different conditions in Table 3.

L240: I would start a new paragraph here

- Thank you for your suggestion. According to this comment, we started a new paragraph in line 274.

L240: Why presented here and not in the first sentence of the paragraph?

- Thank you for your pointed this out. We moved “of the Japanese coastal finless porpoise” to the first sentence in the Result section, as shown in line 261-262.

L242: This was described as a train parameter and not a click parameter. How many samples (# trains) were obtained?

- Thank you for your comment. The click and click train parameters had the same sample size, as mentioned in Table 2 and in line 274-276, “the average ASL and peak frequency at both sites were 174 ± 10 dB re 1 μPa (average ± standard deviation) (n = 316; 172 ± 11 at St. S, n = 136 and 175 ± 9 dB re 1 μPa St. M, n = 180; Table 2), and 134 ± 6 kHz (n = 316; 134 ± 6 at St. S, n = 136 and 134 ± 6 kHz St. M, n = 180; Table 2). ”

L243: Rather belongs in the discussion? Also, having clicks concentrated in the ultrasonic band does not have to imply no masking by ship noise. Even though the majority of ship noise energy is at lower frequencies, it is not limited to that (see e.g. Hermannsen et al. 2014. High frequency components of ship noise in shallow water with a discussion of implications for harbor porpoises (Phocoena phocoena). JASA).

- Thank you for your comment. We have deleted this sentence accordingly.

L245: See comments in Methods on terms for these parameters, and whether they are click or train parameters, or if possibly only data from one click was used for each train.

- Thank you for your observation and suggestion. Accordingly, we have added the following sentences in Materials and Methods in line 199-203, “Click and click train parameters for on-axis echolocation click of finless porpoises were calculated using MATLAB. Click parameters included the apparent source level (ASL) in dB re 1 μPa, peak frequency and center frequency in kHz, -3 dB bandwidth (BW) in kHz, click duration in μs, as well as click train parameters such as ICI in milliseconds (ms) and the number of clicks per train.” and in line 192-193, “T Clicks with the highest amplitude in the series of click trains were analyzed.”

L248: Use the same number of decimals for all numbers on the y axis (should be 1.0, 0.0, -1.0).

- Thanks for your suggestion. We have ensured consistency in decimal placement in the y axis in Fig 2 (1.0, 0.0, -1.0).

L251: See previous comments on whether they were train/click parameters and their terms

- Thank you for your comments. We defined the click and click train parameters, as shown in line 285-289, “Table 2. Summary of finless porpoise echolocation click and click train parameters, and environmental parameters at Seto Inland Sea (St. S) and Mikawa Bay (St. M) Echolocation click parameters (ASL, peak frequency, center frequency, –3 dB BW, and click duration), click train parameters (ICI, number of clicks per train, and range from array), and environmental parameters (temperature, synthetic flow velocity, and noise level).”

L256: Yet one more term (click/source parameter), use consistent terminology.

- Thank you for your comments. We changed this from “source parameters” to “Echolocation click parameters (ASL, peak frequency, center frequency, –3 dB BW, and click duration), click train parameters (ICI, number of clicks per train, and range from array), and environmental parameters (temperature, synthetic flow velocity, and noise level).” as shown in line 287-289. Additionally, we ensured consistency in the use of these terminologies in the manuscript.

L260: As the analyses are centered around the click parameters, and to make it easier to follow between the text and Fig 3, I recommend to align the structure the text and Fig 3 better, starting with the day/night comparison. So, if you want to keep the a-g figures in the current order within Fig 3, begin by saying there was a significant difference between day and night in click duration at both sites (3d), in BW at Site S but not Site M (3e), and in number the of cycles per click at both sites (3f). For the other parameters there was no difference between day and night at any of the sites. And then the same way for the comparison between vessel absence/presence. Make sure to be consistent in the terms of the parameters.

- Thank you for your comments. We have revised Fig. 3 and organized in the results for the click and click train parameters according to the order in Table 2. Additionally, the results changed considerably due to the change in the analysis methods; however, we maintained consistency in the structure of the results, using uniform terminologies.

L270: In the figure this is called "Absent/Present ships", make sure to use consistent terms.

- Thank you for pointing this out. We revised this to “absence/presence of vessel noise” throughout the manuscript.

L283: N (number of samples of each group) is not given anywhere for the comparisons, it should either be added to the figure or listed in a table as commented on the first paragraph of the Results.

- Thank you for pointing this out. For ease of understanding, we have added the number of sample sizes for the different conditions in Table 3.

L289: Could you write the p-values for non-significant results? This in combination with knowing the sample sizes (n) is helpful for judging possible trends/tendencies of non-significant results.

- Thank you for your insightful comment. We changed the analysis method according to the previous comments, but described the p-value of glm and glmm analysis in manuscript for best model. For example, we wrote the results as shown in line 301-305, “For ASL, the best model was the one with the day/night (p = 0.25, S1 Table), absence/presence of vessel noise (p = 0.98, S1 Table), and interaction between the day/night and absence/presence of vessel noise (p < 0.01, S1 Table) as the explanatory variables (AIC = 2326.2, Table 4), performed using GLMM with Residual Maximum Likelihood estimation (REML).” We wrote the sample size in Table 3.

L292: All parameters mentioned previously but center frequency is included in S1 Table, how come?

- Thank you for your comments. We changed S1 Table because the analysis method was changed, based on previous suggestions. A description of the center frequency was not included in S1 Table caption.

L294: The p value is often recommended to be corrected for multiple comparisons, which has not been done here.

- Thank you for your comments. We deleted this sentence because the analysis method was changed, based on previous suggestions. The p-values for GLM and GLMM were included.

L306: Should be "total number (on- and off-axis) regular click trains"?

- Thank you for your suggestion. We changed this part to “A total of 1,257 on- and off-axis click trains (excluding buzz) were recorded at St. S (810 and 447 at day and night, respectively), whereas 4,104 click trains were recorded at St. M (925 and 3,179 at day and night, respectively).” in line 267-269.

L306: As data are analysed per day/night (and vessel absence/presence), and not per hour, how come another approach was chosen here? Why not use the same approach here, i.e. box plots for day/night? Was analysing total clicks and buzzes during vessel noise absent/present beyond the scope of this study? It is a missing piece of the puzzle, so a comment on this in Discussion would be relevant.

- Thank you for your comment. We changed the analysis method and showed the number of on-axis and off-axis click train (excluding buzz) and buzzes. Additionally, we described the ratio of emitting buzz during day and night respectively, as shown in line 267-273: “A total of 1,257 on- and off-axis click trains (excluding buzz) were recorded at St. S (810 and 447 at day and night, respectively), whereas 4,104 click trains were recorded at St. M (925 and 3,179 at day and night, respectively). Additionally, 110 (81 and 29 at day and night, respectively) and 120 buzzes (38 and 82 buzzes at day and night, respectively) were recorded at St. S and St. M, respectively. Moreover, the buzz ratios (percentage of total buzz to the total click train) at St. S and St. M were 8.8% (10.0% and 6.5% at day and night, respectively) and 2.9% (4.1% and 2.6% at day and night, respectively), respectively.”

We only examined whether there was vessel noise or not before and after click events. As there were numerous vessel passages even during times without clicks, it was not easy to count all the ships, and thus the total number of ships at the two sites was beyond the scope of this study and was not reported.

L 312: This is a ratio (=proportion), not rate (e.g. number per hour)

- Thank you for your comment. We changed “rate” to “ratio”.

**Discussion**

In Discussion, the different aspects are presented in another order than in Methods and Results. It facilitates for the reader to follow if the same order us used as far as possible throughout the manuscript.

- Thank you for your comment. We maintained the same order throughout the manuscript to enable the readers to comprehend our idea. Specifically, we inserted the following sub-headings in lines 356, 406, and 436 “Effects of presence of vessel noise on echolocation click and click train parameters”, “Effects of day/night on echolocation click and click train parameters”, and “Comparison of echolocation click and click train parameters with previous findings”.

L319: Note previous questions on click/train parameters

L321: See previous comments om terms

- Thank you for pointing this out. “the click and click train parameters“ was inserted in line 353.

L324: The sub-headings are helpful

- Thank you for your advice. We have added the sub-headings in Materials and Methods, Results, and Discussion sections and maintained the same order throughout the manuscript to make it easier for readers to follow.

L330 See previous comment on that this may be caused by the definition of buzz.

L331: I do not think this can be concluded as the occurrence of buzzes anywhere in the trains have not been investigated, see previous comment on this.

- Thank you for your comment. The definition of buzz was changed according to previous comments as shown in line 180-181, “Five consecutive clicks with ICI < 10 ms was defined as buzz (feeding sound) [13,26]” in Materials and methods. We re-analyzed the all data according to the revised definition.

L333: Between day and night? "Trend" is usually used for non-significant results, but this was significant for all these parameters and both sites, with the exception of BW at Site M. Also see previous comment on whether both t and number of cycles really are needed. L335: See comments above.

L336: "shorter click duration"

- Thank you for your comment. We deleted this sentence because we changed the analysis method, according to the suggestion of the reviewer. The word “trend” was deleted.

L346: duration

L 350: duration

- Thank you for your suggestion. We changed “length” to “duration” throughout the manuscript.

L353: Wouldn't an alternative explanation be that the on average, a higher proportion of the clicks are for echolocation at closer distances at night (which is likely the case if more foraging occurs at night), as less energy would be needed for shorter distances?

L360: I do not understand how this is deducted.

- Thank you for your comment. We removed this section on energy as it was beyond the scope of the results presented in this paper.

L372: Difficult to understand. Do you rather want to say that there was no clear pattern in the response in click parameters to vessel noise across the two sites? However do also consider previous comment on how diel phase and vessel noise may be confounded with the current analyses.

- Thank you for your comment. We deleted this sentence because the analysis method and results were changed. We re-analyzed the data using GLMs and GLMMs to account for the confounding effects of absence/presence of vessel noise and day/night. In the re-analyzed results, ASL was affected by confounding effects of absence/presence of vessel noise and day/night as shown in Fig 3a. Other parameters were not significantly affected by absence/presence vessel noise.

L381: See previous comment on confounding effects, but also, as no difference was observed at Site M, I would say that the sentence is too strongly expressed. It is rather that they may do so (under some circumstances, but not others).

- Thank you for your comment. We re-analyzed the data to account for the confounding effects of absence/presence of vessel noise and day/night, using GLM and GLMM. Additionaly, ASL was influenced by the confounding effect of absence/presence vessel noise and day/night at St. S(Fig. 3a), but not at St. M (Fig. 3a). We discussed the differences between the sites as shown in line 378-405.

L388: I do not understand the logic of this. What do the porpoises gain by increasing the number of pulses, when ASL is reduced? They *can* do so if they have a limited amount of energy to be spent, but what is the advantage of more quieter clicks in a noisy environment?

L389: Clicks per train

- Thank you for your comment. We have removed this sentence because we revised the analysis method, based on the previous suggestion of the reviewer. Absence/presence of vessel noise and day/night did not affect the number of pulses.

L393: Can an increased duration of a click (micro-secs) reduce the risk of masking in a noisy environment?

- Thank you for your comments. We deleted this sentence because we changed the analysis method. The click duration was not affect by the absence/presence of vessel noise as shown in line 320-323, “For click duration, the best model was the one with the day/night (p < 0.01, S1 Table), absence/presence of vessel noise (p = 0.84, S1 Table), the interaction between day/night and absence/presence of vessel noise (p = 0.63, S1 Table), and noise level (p = 0.09, S1 Table) as the explanatory variables (AIC = 2491.7, Table 4), performed using GLMM with REML.”

L396: May also other differences than the number of vessels have caused the different results? A higher number of fishing vessels is usually a result of natural high productivity of fish in the area, and fish species composition, quantity and quality is very likely to affect both presence and echolocation behaviour of porpoises. The hypothesis on vessel impact is only based on general information on numbers of fishing vessels in nearby ports, number of nearby ferry routes, and the results shown in Fig 3 (which may be confounded by diel effects). Also, information on home ranges of the finless porpoise would be useful for evaluation of the impact of the local environment on the likelihood of habituation.

- Thank you for your comments. We deleted this sentence because we changed the analysis method. In the re-analyzed results, there were differences in ASL between the two sites (Fig. 3a). We speculated that the differences between sites could be due to factors, such as familiarity to vessel noise, prey species, and the time of prey species (line 370-401), “At St. S, ASL was significantly affected by the absence/presence of vessel noise and day/night, with a notably higher ASL during the daytime in the absence of vessel noise. In contrast, only slight changes were observed in ASL between the day/night or the presence or absence of vessel noise at St. M. The differences in response between the observation site might be due to various factors, such as habituation to vessel noise, prey species, and other complex environmental factors. For instance, Indo-pacific humpback dolphin (*Sousa chinensis*) in areas with low vessel noise have been shown to respond more strongly to vessels than dolphins in areas with high vessel noise [74]. In the present study, variation in ASL was low at the site with high vessel noise (St. M), whereas variations in ASL was high at the site with low noise (St. S). Therefore, the variation in ASL in finless porpoises could be attributed to different responses to changes in vessel noise, and it is possible that habituation occurred. However, some findings indicate that harbor porpoise do not exhibit habituation to vessel noise, even after living in an environment with high levels of vessel noise [7,75]. Therefore, further studies are necessary to comprehensively elucidate the influence of habituation to vessel noise on click parameters.

In the present study, buzz ratio was higher during the day at the two sites; additionally, there was no significant difference in the number of prey species captured in St. S and St. M. The following prey species of finless porpoise [76] were captured at St. S: whitespotted conger (*Conger myriaster*), Japanease sea bass (Lateolabrax japonicus), east Asian common octopus (*Octopus sinensis*), shrimp, and squid [77]. The prey species captured at St. M were Japanese sardine (*Sardinops melanostictus*), dotted gizzard shad (*Konosirus punctatus*), Japanese anchovy (Engraulis japonicus), Japanese halfbeak (*Hyporhamphys sajori*), red barracuda (*Sphyraena pinguis*), Japanese rockfish (*Sabastes inermis*), whitespotted conger (*Conger myriaster*), Japanese sea bass (*Lateolabrax japonicus*), east Asian common octopus (*Octopus sinensis*), shrimp, and squid [78,79]. Additionally, the following non-prey species of the finless porpoise were captured at both sites: Japanese jack mackerel (*Trachurus japonicus*), red tilefish (*Brachiostegus japonicus*), chubmackerel (*Scomber japonicu*), Japanese spanish mackerel (*Scomberomorus niphonicus*), largehead hairtall (*Trichiurus lepturus*), righteye flounder (Pleuronectidae sp.), and bastard halibut (*Paralichthys olivaceus*), pufferfish (Tetraodonidae sp.), and crab (Brachyura). [77–79]. Furthermore, there were no differences in depth or bottom sediments at both sites. Variations in ASL at both sites may likely not be due to differences in feeding time or prey species.”

Given that other factors might affect the changes ASL, the following sentences were added in line 401-404: “However, since the flow velocity and noise levels between the two sites were different and the porpoises at both sites belonged to different local populations, we can not rule out the possibility that other parameters not examined in this study may have affected the behavior of finless porpoises. Therefore, further studies are necessary to comprehensively elucidate the effect of environmental factors on finless porpoises.”

L396: habituation to?

- Thank you for your suggestion. We deleted this sentence because we changed the analysis method. Additionally, we discussed the effect of habituation to vessel noise (line 375-384), “For instance, Indo-pacific humpback dolphin (*Sousa chinensis*) in areas with low vessel noise have been shown to respond more strongly to vessels than dolphins in areas with high vessel noise [74]. In the present study, variation in ASL was low at the site with high vessel noise (St. M), whereas variations in ASL was high at the site with low noise (St. S). Therefore, the variation in ASL in finless porpoises could be attributed to different responses to changes in vessel noise, and it is possible that habituation occurred. However, some findings indicate that harbor porpoise do not exhibit habituation to vessel noise, even after living in an environment with high levels of vessel noise [7,75]. Therefore, further studies are necessary to comprehensively elucidate the influence of habituation to vessel noise on click parameters.”

L399: of what?

- Thank you for your comment. We removed this sentence because we revised the analysis method.

L399: habituation to?

- Thank you for your suggestion. We deleted this sentence because we changed the analysis method. We have added information about habituation to the vessel noise as shown in line 375-384, “For instance, Indo-pacific humpback dolphin (*Sousa chinensis*) in areas with low vessel noise have been shown to respond more strongly to vessels than dolphins in areas with high vessel noise [74]. In the present study, variation in ASL was low at the site with high vessel noise (St. M), whereas variations in ASL was high at the site with low noise (St. S). Therefore, the variation in ASL in finless porpoises could be attributed to different responses to changes in vessel noise, and it is possible that habituation occurred. However, some findings indicate that harbor porpoise do not exhibit habituation to vessel noise, even after living in an environment with high levels of vessel noise [7,75]. Therefore, further studies are necessary to comprehensively elucidate the influence of habituation to vessel noise on click parameters.”

L400: Also worth mentioning here that Dyndo et al. 2015 did not find any sign of habituation, and Wisniewska et al. 2018 also discuss that habituation did likely not happen.

- Thank you for your comment. We deleted this sentence because we changed the analysis method, but we added the reference recommended by the reviewer and discussed the possibility of habituation to vessel noise (line 380-382), “However, some findings indicate that harbor porpoise do not exhibit habituation to vessel noise, even after living in an environment with high levels of vessel noise [7,75].” The references 7 and 75 are for Dyndo et al. (2015) and Winsniewska et al. (2018), respectively.

L400: Is this relevant when you have not looked at echolocation rate in relation to noise levels/vessel presence?

L403: Can you support this statement by using your sound measurements?

L405: Delete. Diel cycle is discussed under the previous heading and there are no other results to discuss here.

- Thank you for your comments. We have removed this sentence because we changed the analysis method, based on previous suggestions. Consistent with your observation, we did not check echolocation rate in relation to noise levels/vessel presence.

L424: See Kyhn et al. 2013 Clicking in a killer-whale habitat... PlosOne for a discussion on that porpoises seem to have reached the upper frequency limit for functional biosonar in water.

- Thank you for your comments. We have deleted this sentence because we changed the analysis method; however, we have cited the study recommended by the reviewer and described the limitation of fluctuating frequencies (line 425-435), “In the present study, there was no significant difference in peak frequency and center frequency between day and night. Although the center frequency of melon-headed whale has been shown to increase at night [18], the frequency bands emitted by melon-headed whale and finless porpoise are different. Specifically, finless porpoises emit echolocation clicks at frequencies of 125–135 kHz [21,29-31], whereas melon-headed whale produce echolocation clicks at frequencies of 25–30 kHz [83], which is a difference of approximately 100 kHz. Therefore, the fluctuation of frequency changes caused by surrounding environment of finless porpoise and melon-headed whale could be attributed to differences in frequency band. Additionally, a previous study indicated that NBHF species are less likely to exhibit fluctuations in frequency [84]. The range of fluctuation in frequencies in the present study was small, necessitating further studies with more data for NBHF species.” Reference [84] is for Kyhn et al. (2013).

L424: or

- Thank you for your comment. We removed this sentence because we revised the analysis method, based on previous suggestions.

L438: See Hermannsen et al. 2014 JASA: High frequency components of ship noise in shallow water with a discussion of implications for harbor porpoises (Phocoena phocoena)

- Thank you for your comments. As indicated, it is possible that the echolocation clicks of porpoises and dolphins could be masked by the high-frequency components of vessel noise. In consideration of this point and changes in the analysis method, we removed the section described in L438.

L454: Have you considered the study by Sørensen et al. 2018 Sci Rep on characteristics of click communication of harbour porpises?

- Thank you for your comments. We removed this sentence because we revised the analysis method, based on previous suggestions.

L456: See also previous comment on how buzz was defined

- Thank you for your comment. We have revised the definition of buzz, as shown in line 180-181, “Five consecutive clicks with ICI < 10 ms was defined as buzz (feeding sound) [13,26]”.

L459: This comes a bit surprising (why start talking about differences among species in a discussion on diffidences between two sites?). Suggest rephrasing so it becomes clear from the start that you want to discuss that the differences between the sites may be due to the three factors that you mention.

- Thank you for your comment. We have removed this sentence and the discussion about species, as it was beyond the scope of this study.

L465: Have you considered Galatius et al. 2012 on skull morphology of harbour porpoises, hypothesised to be an evolutionary effect of different foraging habitats? I.e. differences in skull morphology may be a sign of both different click characteristics and acoustic behaviour.

- Thank you for suggesting this study. Accordingly, we have added information on skull morphology of harbor porpoise (line 446-447), “Moreover, the skull morphology of harbor porpoise exhibits evolutional adaptation to prey species [87].”

**Conclusion:**

L473: I recommend to shorten Conclusions to a minimum, this should only contain the absolute essence of the findings of the study, preferably only a few sentences.

Please do also keep previous comments on terminology, and the unbalanced/confounded analyses of day/night and vessel noise absence/presence, and other differences than vessel traffic between the sites, that may have affected the results.

- Thank you for your comment. Based on the revised analysis method and the results obtained from it, we modified conclusion. Additionally, we organized the discussion in the order of the results, with relevant subheadings. The revised conclusion is shown in line 456-465, “In the present study, echolocation click and click train parameters were influenced by the day/night. Specifically, ASL was higher in the absence of vessel noise during the daytime at the site with low vessel traffic. Additionally, finless porpoise increased their resolution and amount of sound information by shortening the click duration, increasing -3 dB BW, and reducing ICI at night. Overall, these findings contribute to our understanding of species adaptation in response to day/night change. To accurately assess the impact of vessel noise on echolocation characteristics, it is important to consider day/night factors. However, only acoustic monitoring was performed in this study, and factors, such as the behavioral state of vocalizing individuals and the speed and size of vessels were not considered, indicating the need for further studies on the effects of these factors on echolocation.”

L476: click duration

- Thank you for your comment. We changed “t” to “click duration” throughout the manuscript.

L481: Was there really such a difference, and how is can reduced ASL and increased click duration make sense as a vigilance behaviour?

Thank you for your comment. We removed this sentence because we revised the analysis method, based on previous suggestions.

L484: See previous comment with reference to Kyhn et al. 2013 supporting that they are not capable of changing the frequency

- Thank you for your insightful comment. We have removed this sentence from the conclusion to make it concise. Additionally, the effect of vessel noise and day/night on frequency was highlighted in the Discussion section, citing Kyhn et al. (2013), as shown in line 433-434, “Additionally, a previous study indicated that NBHF species are less likely to exhibit fluctuations in frequency [84].” in Discussion. Reference [84] is Kyhn et al. (2013).

L491: Or was it confounded?

- Thank you for your comments. We removed this sentence because we revised the analysis method based on the previous comments. We added information regarding the confounding effect of absence/presence of vessel noise, day/night, and environmental factors, based on the results of GLM and GLMM analysis. Notably, ASL was affected by confounded effect between absence/presence of vessel noise and day/night (Fig. 3). Additionally, we observed that click and click train parameters were more influenced by day/night than by the abnsece/presence of vessel noise, and this was indicated in line 461-462:“To accurately assess the impact of vessel noise on echolocation characteristics, it is important to consider day/night factors”.

------------------------

Reviewer 2 reports:

General comment:

I found this manuscript hard to follow because of grammatical issues. Many grammar mistakes make the article unclear and a grammar check by a native English speaker is definitely required. I mentioned some of the mistakes in my comments, but these are just examples, and the full paper should be corrected.

- Thank you for your comments. The manuscript was reviced based on feedback from two reviewers, with grammatical errors corrected. Additionally, it was edited by native English speakers (Editage).

In the manuscript, the gap in the literature is unclear. As a reader, I am not able to understand what has been done on this species and other odontocetes in previous studies and therefore what is new here (except the location).

- Thank you for your comment. The introduction has been extensively edited to indicate the gap in literature and confirm the novelty of the study (line 42-57): “Over the years, several studies have been performed to elucidate the impact of vessel noise on echolocation clicks. For instance, Atlantic bottlenose dolphins (*Tursiops truncatus*) and harbor porpoises (*Phocoena phocoena*) decreased emitting buzz rate, feeding trial sound and one of the echolocation click [13,14], in response to the presence of vessels, indicating a decrease in their feeding activity [15,16]. Additionally, Lahille’s bottlenose dolphins (*T. truncatus gephyreus*) emitted significantly fewer echolocation clicks in the presence of vessels in ~ 250-m radius [17]. Moreover, melon-headed whales (*Peponocephala electra*) increased echolocation click source level in correlation with the level of ambient sound pressure [18]. However, previous studies were mainly focused on the effects of vessel noise on the echolocation click train emitting rate, with limited studies on variations in echolocation click and click train parameters like the study of Baumann-Pickering et al. [18]. In contrast, the effects of vessel noise on whistle parameters have been extensively studied. For example, common bottlenose dolphins were observed to shift whistle frequency [19] and increase sound pressure in the presence of vessels [20]. Similarly, studies are necessary to identify echolocation parameters that are affected by vessel noise, especially in small cetaceans to improve conservation efforts.”

The material and methods part is very hard to follow because of grammar, repetitions, and bad organization.

- Thank you for your comments. Grammatical, structural errors were extensively addressed during the revision. Moreover, the entire manuscript has been edited by two reviewers and native English speakers.

Discussion overall contains good hypotheses, except for the difference between the two sites that focus on some potential explanations and forget some others. Moreover, some studies conducted on captive porpoises (asiaeorientalis and sunameri) were never cited while they could be useful to interpret some of your results.

- Thank you for your comment. The results and discussion were substantially reorganized due to a change in the statistical analysis method and changes in the results. Subheadings were added and presented in the same order to make it easier for the reader. Additionally, we referenced previous studies on captive porpoises (line 428-429), “finless porpoises emit echolocation clicks at frequencies of 125–135 kHz [21,29-31]”

In addition, no limitations are mentioned. The dataset is very small, and this should be mentioned, especially since the results given are surprising.

- Thank you for your comment. To address concerns about the small sample size, we conducted additional analysis to increase the sample size. Then, we described the limitation and results of this study in the conclusion, as shown in line 462-465, “However, only acoustic monitoring was performed in this study, and factors, such as the behavioral state of vocalizing individuals and the speed and size of vessels were not considered, indicating the need for further studies on the effects of these factors on echolocation.”.

Specific comments:

**Title:**

is it grammarly correct to say “in response to diel”? I believe it is not, better find another wording.

- Thank you for your comments. Accordingly, the title has been revised to “Variations in echolocation click characteristics of finless porpoise in response to absence/presence of vessel noise and day/night”.

**Abstract:**

L16: navigate in their environment

- Thank you for your observation. This has been corrected to “to feed and navigate” in line 16.

L17: “making it… an important role” is not correct grammatically speaking

- Thank you for your comment. This has been corrected to “making it an essential function for their survival” in line 16-17.

L22: where?

- We added more explanations to the text “at two sites in Japan: Seto Inland Sea and Mikawa Bay” in line 23-24.

L22: “the effects of diel response” is not grammatically correct

L24: diel cycle of visitation? Feeding sound rate? What does this mean?

L25: responses were not constant for the same species? You actually studied a single species, so no need to mention this. And what does “constant” mean?

- Thank you for your comments and suggestions. This sentence has been deleyed because we revised the analysis method, based on previous comments by another reviewer.

L27: per click train

- Thank you for your suggestion. “number of pulses in a click train” was changed to “number of clicks per train” throughout this manuscript according to the comments from Reviewer 1.

L27-28: during the minute

L29-30: bad wording. I suggest “In contrast, in the area subject to more boat traffic, click length and number of cycles per click decreased and source-level increased before and after vessel traffic noise.”

- Thank you for your suggestion. This sentence has been deleted because we changed the analysis method, based on the previous comments by another reviewer. However, the grammatical errors are notedt.

**Introduction:**

L40: their characteristics (no need to repeat “echolocation clicks”)

- Thank you for your comment. This sentence has been deleted because we changed the analysis method, based on the previous comments by another reviewer. We were careful not to repeat the same words constantly.

L44: I don’t think you can speak of acoustic observations since it is not something visible. Use “recording” instead.

- Thank you for your comment. This sentence has been deleted because we changed the analysis method, based on the previous comments by another reviewer. Acoustic monitoring or recording were used interchangeably throughout the manuscript.

L43-45 and 46-48: these two sentences are saying opposite things… you tell us that most studies did not study diel rhythm and then you state that diel rhythm has been studied a lot through PAM…

- Thank you for your comment. This sentence has been carefully edited without altering the intended meaning. Some studies reported that echolocation characteristics changed depending on the diel cycle as shown in line 60-64, “For instance, melon-headed whales exhibit higher center frequencies at night owing to increased feeding activity [18], and harbor porpoises emitted high proportion of click trains with short inter-click intervals (ICIs) to forage or investigate objects at close range [22]. Additionally, the diel behavior of harbor porpoises likely depends on changes in the diel behavior of their prey [23–26]”. Additionally, we added the sentence about knowledge gaps as shown in line 64-66, “However, studies on the variations in echolocation click characteristics during the day/night have not been conducted in several species, and it is unclear if diel variations in echolocation click are associated with vessel noise. ”

L54-56: you just gave examples that say exactly this… I suggest removing this sentence or placing it before the examples.

- Thank you for your comments. We have removed this sentence, as per your suggestion.

L65: you first speak about the effect on vocalizations and then give an example about energetic budget and then speak about vocalizations again… I cannot see the usefulness of this example here.

- Thank you for your comments. We have removed this sentence accordingly.

L72: is necessary

- Thank you for your suggestion. This sentence was deleted because we revised the introduction according to the comments from Reviewer 1.

L74-83: you are mixing information on two subspecies. If your goal is to state that there has been research on this topic for asiaeorientalis but not for sunameri, state it clearly and don’t mix information between these two subspecies.

- Thank you for your comment. We revised it to cleary state the information for each of the two subspecies, along with the references to avoid confusion. Specifically, the two variants were discussed, as shown in line 78-82 “Recently, some studies have examined the impact of vessel noise on Yangtze finless porpoise. For example, increase in vessel noise have been shown to affect Yangtze finless porpoise distribution [36,37], cortisol levels [38], and activity [39]. Additionally, Yangtze finless porpoise emit more buzz at night for foraging [35,40], finless porpoises avoid passing boats by diving [41].”

L88-91: it would be interesting to know what the other studies of clicks on the same species found, even if it is in different locations.

- Thank you for your comment. The introduction has been edited to take care of redundancies. Additionally, findings on finless porpoise at other sites (Bohai Sea and Taiwan Strait) were highlighted in in the Disucussion section, as shown in line 437-453 “In the present study, click and click train parameters of wild finless porpoise in Japanese coastal areas were measured. Compared with the click and click train parameters of the finless porpoise in the Liao-dong wan Bay, Bohai Sea [29], and the Taiwan strait [43], finless porpoise examined in the present study had a higher peak frequency (Liao-dong wan Bay: 121 ± 4, Taiwan strait: 133 ± 4), wider bandwidth (Liao-dong wan Bay: 18 ± 3, Taiwan strait: 10 ± 2), and shorter click duration (Liao-dong wan Bay: 80 ± 11, Taiwan strait: 112 ± 8) [29,43]. These differences were more significant than those between St. S and St. M and may be caused by factors such as skull morphology [44,46], environmental characteristics, and behavior. In some bat species, skull morphology has been suggested to be associated with echolocation parameters [85,86]. Moreover, the skull morphology of harbor porpoise exhibits evolutional adaptation to prey species [87]. However, it is unclear whether there are differences in skull morphology between finless porpoises in Liao-dong wan bay, Taiwan strait, and the Japanese coastal area. Therefore, further studies are necessary to elucidate the relationship between skull morphology, click and click train parameter, and environmental factors. Harbor porpoise exhibited variations in click train parameters for communication [88]. Thus, it is necessary to examine whether variations in click and click train parameters are dependent on the behavioral state of finless porpoise.”

L86-91: this information belongs to the previous paragraph, it should be given before you state what you did in your study.

- Thank you for your suggestion. We have revised the sentences, as shown in line 85-91: “Echolocation click of wild finless porpoise have been reported in the coastal waters of mainland China, Liao-dong-wan Bay in the Bohai Sea, and the western coast of the Taiwan Strait [42,43]; however, such study has not been performed on wild finless porpoise in Japanese coastal areas. Wild finless porpoises are genetically and morphologically divided into five populations [44–46], each of which is highly vulnerable to anthropogenic impacts due of low level of genetic diversity [47]. Therefore, understanding the impacts of vessel noise is necessary for the conservation of the finless porpoise in the Japanese coastal areas.”

**Matériel and methods:**

L97: acoustic recording

- We revised “Acoustic observations” to “Acoustic recording” in line 103.

L101: boats

- We changed “boat” to “boats” in line 107 and 108.

L124-125: a total of six days of recording for each area? That is not what I understand from table 1…

- Thank you for your observation. The aim was to conduct acoustic observations multiple times for ~8 days; however, for clarity, the recording duration has been listed in Table 1.

L128: was not able

- We changed this to “was not able”.

L130: in the click train of the echolocation? What does it mean?

- Thank you for your observation. This sentence “in the click train of the echolocation” means “a series of click trains”. This has been revised to “of the consecutive clicks” in line 143, as per the suggestion provided by Reviewer 1.

L134: was used to discriminate?

- Thank you for your comment. Yes, we used the sound pressure ratio of the two microphones of A-tag to classify sounds between Delphinidae and Phocoenidae. We revised the sentence as shown in line 146-147, “and the sound pressure ratio was used to differentiate between Delphinidae and Phocoenidae [52,53]”.

L126-139: overall very unclear, and hard to understand, mostly because of the bad grammar.

- Thank you for your comment. We revised this part to make it clearer and more understandable. The English grammar has been checked and edited by a native speaker at Editage company as shown in line 139-151: “The A-tag is a stereo event recorder of pulse information capable of recording the time, sound pressure levels of each of the two hydrophones, and the relative azimuth of a pulsed sound that exceeds a set sound pressure threshold. The A-tag was not able to record the frequency information, but the echolocation click train was detected by using smooth changes in sound pressure and the ICI of the consecutive clicks [35,50,51]. The two hydrophones of the A-tag had different frequency responses (maximum sensitivities at 130 and 70 kHz) and were placed 190 mm apart. The time difference between the arrival of the sound from the two hydrophones was measured to determine the relative azimuth, and the sound pressure ratio was used to differentiate between Delphinidae and Phocoenidae [52,53]. The distance to the sound source from the array was calculated using the relative azimuth values and a trigonometric function [54,55]. The bandpass filter in the A-tags was adjusted to 55–235 kHz, and amplification was modified to + 60 dB. The sampling rate was set to 0.5 ms and the amplitude threshold to 360 counts (approximately 6.3 Pa).”

After reading this part, it is not clear to me how the acoustic data was collected. The collection period is around 2 months in table one, for a total of 638h for the first location for instance, and the text mentions 6 days of data. So, how was the data sampled? Was it 1-minute recording each hour for example?

- Thank you for your observation. We collected data by conducting muitiple rounds of continuous recordings for up to 8 days at each sites until the battery was exhausted. For clarity, the recording duration has been listed in Table 1.

L160: you should say that “a wave with an interval of less than 1ms from the previous was discarded…”

- Thank you for your comment. We added the sentence, “while reflected waves (i.e. waves within < 1 ms interval of the previous wave) were excluded [50].” in line 172-173, as you suggested.

L165: what exactly was checked?

- Thank you for your comment. We checked manually each data on the softwre to see whether there were any data with characteristics of the finless porpoise echolocation clicks. We revised this sentence to “Each click train detected was visually inspected to confirm whether it met the criteria for a click train, using Igor software, according to the procedures described by Akamatsu et al. [33, 50] and Kimura et al. [51].” in line 176-178.

L170: remove “was”

- We removed “was”.

L172: click trains with constant amplitudes or other amplitude patterns were not included?

- Thank you for your comment. On-axis echolocation clicks without noise are characterized by a small amplitude at the beginning, which gradually increases, and finally decreases as reported in previous studies. Since this study focused the characteristics of on-axis echolocation clicks, wavelets with other amplitude patterns were excluded because these are off-axis echolocation clicks. This has been explained in line 189 “the amplitude of the series of click trains should first increase and then decrease (*sensu* [61])” and in line 192-193, “Clicks with the highest amplitude in the series of click trains were analyzed”.

L173: the (4) does not give any criteria, what should the maximum amplitude be like?

- Thank you for your comment. This sentence in line 192-193 has been in revised to read “Clicks with the highest amplitude in the series of click trains were analyzed.”

L176-178: what about criteria 4 and 5?

- Thank you for for your suggestion. We added (4) and (5) in line 192-193, “Criterion (1) was analyzed using the A-tag and SoundTrap data, (2) and (6) were analyzed using the A-tag data, and (3), (4), and (5) were analyzed using the SoundTrap data.”

L197: salinity data was obtained from temperature data?

- Thank you your observation. We added explanation in line 210-214, “Salinity data at St. S were obtained from the Hirae Iwakuni fixed-line survey conducted by the Inland Sea Research Department of the Yamaguchi Prefectural Fisheries Research Center at 33°50’12″N, 132°02’04″E (~ 7.7 km from St. S) once a month during the same period, using the Yamaguchi Prefecture submarine and fisheries research vessel “Seto” [64].”

L201-203: a good example of an explanation that is very unclear because of grammar. You should write: “The ICI was the time interval between consecutive pulses of a click train.” I don’t understand if you speak about the amplitude of a click or the difference of amplitude between two clicks, therefore I cannot even reformulate your full sentence…

- Thank you for your comment. This sentence was also pointed out by Reviewer 1. We revised this to “ICI was calculated as the average interval between two ICIs before and after the click with the maximum amplitude” in line 224-225, as suggested by Reviewer 1.

L228: produced by porpoises

- This was corrected to “produced by the finless porpoise” as shown in line 179.

L231: why is the day/night separation fixed like this when you stated earlier that you used the sunset/sunrise data? It is not consistent…

- Thank you for your comment. We re-analyzed the data according to the comments of Reviewer 1 and categorized day/night in a manner similar to the day/night categorization of the click and click train parameters, as shown in line 182-183 “Additionally, click trains and buzzes were categorized as day/night [56,57], and their hourly average was calculated”.

**Results:**

L234-235: only that little clicks? Are you sure you don’t mean click trains? Some click trains contain more clicks than this…

- Thank you for your comment. We examined both click and click train parameters. We revised the manuscript to clearly state the difference between click and click train parameters as shown in line 199-200, “Click and click train parameters for on-axis echolocation click of finless porpoises were calculated using MATLAB. Click parameters included the apparent source level (ASL) in dB re 1 μPa, peak frequency and center frequency in kHz, -3 dB bandwidth (BW) in kHz, click duration in μs, as well as click train parameters such as ICI in milliseconds (ms) and the number of clicks per train” in Materials and methods.

Table 2: you need to provide details for the stat tests, for example, the exact p-value and the U for Mann Whitney.

- Thank you for pointing this out. We added the exact p-value in Table 2 and analysis method in the caption of Table 2 as shown in line 289-291, “ASL was compared using Student’s t-test, while the other parameters were compared using Mann–Whitney U-test to determine significant differences between the sites.”

L269-281: why do we have a table for inter-site differences, but everything is written in the text for other differences? I recommend staying consistent in the way you present the same kind of results. I recommend being careful with the words increase and decrease. You can say that ASL was higher after a boat noise for instance, but saying that ASL increases after a boat pass by has a more specific meaning, thy does not correspond to the analysis you did.

- Thank you for your comments. We have revised the manuscript for consistency. We determined the effect of absence or presence of vessel noise by listening to the sounds one minute before and after the production of on-axis sound by finless porpoise, as shown in line 228-229 “The absence/presence of vessel noise was determined by manually listening to the sounds 1 min before and after on-axis click train detection” in Materials and methods.

L306-313: no stats here? You did not analyze the buzz ratio differences between night and day?

- Thank you for your comments. Based on your observation, we have included details of on-axis and off-axis click trains and number of buzzes during the day and night, and indicated buzz ratio (line 267-273), “A total of 1,257 on- and off-axis click trains (excluding buzz) were recorded at St. S (810 and 447 at day and night, respectively), whereas 4,104 click trains were recorded at St. M (925 and 3,179 at day and night, respectively). Additionally, 110 (81 and 29 at day and night, respectively) and 120 buzzes (38 and 82 buzzes at day and night, respectively) were recorded at St. S and St. M, respectively. Moreover, the buzz ratios (percentage of total buzz to the total click train) at St. S and St. M were 8.8% (10.0% and 6.5% at day and night, respectively) and 2.9% (4.1% and 2.6% at day and night, respectively), respectively.”

**Discussion:**

L319: of on-axis

- Thank you for your observation. It has been corrected to “characteristic of on-axis” in line 352.

L325: parameters

L326: click trains and buzzes

- Thank you for your comments. This sentence has been deleted because we revised the analysis method based on the previous comments by the reviewer 1.

L327: don’t refer to figures in the discussion

- Thank you for your comments. Accordingly, we have deleted figure and table citations in the discussion.

L327: is it a higher presence or a higher click emission? Porpoises could be there and emit much fewer clicks because of some diurnal changes in behavior or because of human activities.

L330: same

- Thank you for your comments. This sentence was deleted because we revised the analysis method based on the previous comments of the reviewer 1, and the new results were discussed appropriately. In the revised manuscript, this data was used to indicate differences buzz ratio between day and night, as shown in line 385, “buzz ratio was higher during the day at the two sites.”

L333: why use “although” here? There are no contradictions between the two parts of the sentence…

L343-344: which changes? Same as yours?

- Thank you for your comments. We have deleted these sentences in the discussion and result sections because we changed the analysis method.

L344-345: I am not currently reading a lot about this topic, but it surprises me a lot that only one study investigated diel changes in clicks in odontocetes… I believe there are studies on Yangtze finless porpoises at least, and probably on bottlenose dolphins too. I suggest that you make sure you cited all the relevant literature.

- Thank you for your comments. Studies on vocalization or variations in echolocation rate during the day and night have been performed on many small cetaceans. Yangtze finless porpoise, bottlenose dolphin, and harbor porpoise were observed for diel changes of echolocation clicks. However, to the best of our knowledge, diurnal change or variation in click or click train parameters has only been conducted on melon-headed whales. This was addressed in both the Introduction and Discussion sections.

L358: remove “exhibit”

- Thank you for your comments. We removed this sentence because we revised the analysis method based on the previous comments by reviewer 1.

L350-357: here, you cite some literature about diel changes in odontocetes, but in the previous paragraph, you say that there are almost no studies about this… Please be clear on what points are new in your study and have not been investigated yet, because for now, it looks like you overstate the novelty of your study.

- Thank you for your comments. We deleted this sentence because we changed the analysis method. However, the previous studies about diel changes in echolocation clicks are cited in line 59-68 in the introduction “diel changes in echolocation click characteristics have been observed in some species. For instance, melon-headed whales exhibit higher center frequencies at night owing to increased feeding activity [18], and harbor porpoises emitted high proportion of click trains with short inter-click intervals (ICIs) to forage or investigate objects at close range [22]. Additionally, the diel behavior of harbor porpoises likely depends on changes in the diel behavior of their prey [23–26]. However, studies on the variations in echolocation click characteristics during the day/night have not been conducted in several species, and it is unclear if diel variations in echolocation click are associated with vessel noise. Therefore, it is necessary to examine variations in echolocation click during the day/night and in the absence/presence of vessel noise.” We revised this sentence because we changed the analysis method, and the results and discussion were modified accordingly.

L365: suggest

L364-369: why is this information given separately from the other paragraphs? The argument is the same…

- Thank you for your comments. The discussion and results section was heavily revised due to a change in the analysis method, and this sentence was deleted.

L388: how can an increase in vigilance result in a lower ASL? Would they consider boats as predators and therefore be careful not to be detected by this potential predator? Or do you have another hypothesis? This result is really weird compared to previous studies. if true, it would mean that finless porpoises have a different strategy against noise than other odontocetes and that they may be impacted more since it is impacting their communication and echolocation much more. I would like a more detailed discussion on this point. I would also like you to discuss a bit the habituation process.

- We appreciate your observation. The results obtained after our re-analysis also showed that ASL was higher in the absence of vessel noise during the day at St. S, which was contrary to previous findings. We hypothesized that the decrease in ASL might be due to vigilance in response to vessel noise, similar to the reduced vocalization in the presence vessel traffic or predator. Accordingly, so additions have been made to justify this result in the discussion sectionin lines 362-367, “In contrast, some studies showed that the vocalization rate of echolocation clicks decreases in the presence of vessels due to acoustic interference and enhanced vigilance [15,72]. Moreover, cetaceans have been shown to exhibit similar responses to predators and vessel noise [73]. Therefore, it is possible that the observed decrease in ASL in response to vessel noise in the present study may be attributed to vigilance behavior in the presence of vessels. ”

Although the effect of habituation remains unclear, we cited previous studies that indicated behavior changes in response to vessel traffic (line 375-377), “For instance, Indo-pacific humpback dolphin (*Sousa chinensis*) in areas with low vessel noise have been shown to respond more strongly to vessels than dolphins in areas with high vessel noise [74]”

L389-390: it would have been interesting to analyze clicks after the noise to see when parameters come back to “normal”

- Thank you for your comments. We agree that it would have been interesting to analyze clicks after the noise. However, it was not possible to identify individuals based on the sound in this study; therefore, it would be difficult to determine the source of echolocation click and click train parameters after vessel noise has ended. However, we intend to identify individuals by visual observation in future studies.

L391-395: such a big difference between the two populations. If these data were mine, I would

- Thank you for your comment. Re-analysis showed that the only difference in response between St. S and St. M was ASL. Specifically, there was a significant difference in ASL between the sites (Table 2), which could be attributed population differences. However, differences in response to vessels and day/night could be due to other ambient and other factors. The two populations are widely distributed, and since our study was conducted at one site per time, we would like to elucidate whether there were differences between the two populations in future studies.

L406: habituate is a verb, not a noun, you cannot use it like this. Check elsewhere

- Thank you for your comments. The discussion section was heavily revised due to change in the analysis method. Accordingly habituate was replaced with “habituation” in line 374 in the discussion section.

L429: present study

- Thank you for your comments. The discussion section was heavily revised due to a change in the analysis method and results, and this sentence was deleted.

L372-441: discussion elements are present, but like elsewhere, some problems with grammar and organization make it hard to follow.

- Thank you for your comments. The discussion section has been edited by native English speakers to improve the structure and grammar.

L446-449: we are in the part about the difference between sites, why mentioning again day and noise effects? Keep this for the conclusion.

- Thank you for your comments. The discussion section was heavily revised due to change in the analysis method, and these sentences were deleted.

L455-457: or they may have different feeding strategies!

- Thank you for your comments. We did not consider the effect of feeding strategies; however, we examined the effect of feeding behavior indirectly by determining buzz ratio, and as shown in line 385: “buzz ratio was higher during the day at the two sites”.

L457-458: what does it mean? Were porpoises visually observed during all the recordings? I don’t think so. The only thing that could explain this is habitat use: maybe you put your array in a feeding ground for a site and in a traveling area for the other site. But there are many other hypotheses like the different feeding behavior, or the different prey density.

- Thank you for your comments. This sentence has been deleted because of the change in analysis method based on the previous comments by the reviewer. In the present study, visual observations were not conducted, and as you indicated, there may be other factors influencing click and click train parameters. Accordingly, information on prey species were included in line 385-399, “additionally, there was no significant difference in the number of prey species captured in St. S and St. M. The following prey species of finless porpoise [76] were captured at St. S: whitespotted conger (*Conger myriaster*), Japanease sea bass (*Lateolabrax japonicus*), east Asian common octopus (*Octopus sinensis*), shrimp, and squid [77]. The prey species captured at St. M were Japanese sardine (*Sardinops melanostictus*), dotted gizzard shad (*Konosirus punctatus*), Japanese anchovy (*Engraulis japonicus*), Japanese halfbeak (*Hyporhamphys sajori*), red barracuda (*Sphyraena pinguis*), Japanese rockfish (*Sabastes inermis*), whitespotted conger (*Conger myriaster*), Japanese sea bass (*Lateolabrax japonicus*), east Asian common octopus (*Octopus sinensis*), shrimp, and squid [78,79]. Additionally, the following non-prey species of the finless porpoise were captured at both sites: Japanese jack mackerel (*Trachurus japonicus*), red tilefish (*Brachiostegus japonicus*), chubmackerel (Scomber japonicu), Japanese spanish mackerel (*Scomberomorus niphonicus*), largehead hairtall (*Trichiurus lepturus*), righteye flounder (Pleuronectidae sp.), and bastard halibut (*Paralichthys olivaceus*), pufferfish (Tetraodonidae sp.), and crab (Brachyura). [77–79].”

Figure 1: I suggest adding the name of each instrument to the D figure.

→ Thank you for your comment. We have added the name of each instrument.

Figure 5: Y-axis title missing

→ Thank you for your comment. Figure 5 was removed in the revised manuscript owing to the fact that the change in analysis method, as suggested, altered the results.
