# Supplementary material for: Variations in echolocation click characteristics of finless porpoise in response to day/night and absence/presence of vessel noise
Source: PLoS One. 2023 Aug 4;18(8):e0288513. doi: 10.1371/journal.pone.0288513 (PMC10403093; doi:10.1371/journal.pone.0288513)
Supplement: Attachment — Submitted filename: response to Reviewers.docx [file pone.0288513.s006.docx]

Thank you for dedicating your time reviewing and thoroughly assessing our manuscript. We sincerely appreciate your valuable feedback, which has greatly contributed to the improvement of our work. We have carefully addressed the journal’s requirements and diligently incorporated the suggestions provided by the reviewers. In the revised manuscript, we have used the following color scheme: black to denote the journal requirements and reviewer’s comments, blue to indicate our responses, and green to highlight the revised words/sentences.

-------------------------------------------------------------------------------------------------------

Academic Editor Requirements:

I agree with reviewer 3’ comment that concerns about statistical analysis need to be addressed. Prior to using GLM, you examined the differences of parameter values between the sites by t-test or U-test. However, these datasets can be biased in the sampling process (e.g. one site may have more samples taken during the day, while another site may have fewer samples taken at night). In such cases, simple t-test and U-test are not suitable for examining differences. I suggest using GLMM instead of GLM for all statistical models. Alternatively, as reviewer 3 suggested, you could apply GLM including “study site” as an additional explanatory variable.

 Thank you for your comments. We appreciate the suggestions provided by both editor and reviewer 3. Based on these comments, we revised the statistical analysis approach. Instead of using t-test or U-test to examine the differences between sites, we employed GLMs to include “study site” as an additional explanatory variable as the reviewer 3 suggested. With these modifications and the responses to reviewer's comments, although there were minor revises, the overall results remained largely unchanged. Therefore, we did not make any changes to the figures and conclusions.

In discussion, you can mention about the difference between the study sites (especially with regard to Fig. 3A). But since you did not examine the effect of the sites by statistical tests, you should tone down the message (line 356-358, 369-370 and maybe 456-457). The use of “significantly” is not suitable.

 Thank you for your comments. According to the comments, we toned down the messages avoided the use of the term “significantly.” The modified sections are as follows: line 341–342: “ASL was higher during the day in the absence of vessel noise at St. S than under other conditions”, line 360–362: “At St. S, ASL was affected by the absence/presence of vessel noise and day/night, with a notably higher ASL during the daytime in the absence of vessel noise”, and line 439–440: “ASL was higher in the absence of vessel noise during the daytime at the site with low vessel traffic”.

In this paper, vessel noise did not significantly affect echolocation click and click train parameters (according to the abstract). If so, you should tone down the phrase “absence/presence of vessel noise” in the title. The possible title could be “Variations in echolocation click characteristics of finless porpoise in response to day/night and absence/presence of vessel noise”.

 Thank you for your comments. As you pointed out, the echolocation click and click train parameters of finless porpoises were indeed more influenced by day/night rather than absence/presence of vessel noise. Therefore, we made the necessary revision as per your suggestion, titled “Variations in echolocation click characteristics of finless porpoise in response to day/night and absence/presence of vessel noise.”

Journal Requirements:

Please review your reference list to ensure that it is complete and correct. If you have cited papers that have been retracted, please include the rationale for doing so in the manuscript text, or remove these references and replace them with relevant current references. Any changes to the reference list should be mentioned in the rebuttal letter that accompanies your revised manuscript. If you need to cite a retracted article, indicate the article’s retracted status in the References list and also include a citation and full reference for the retraction notice.

 Thank you for your comment. We thoroughly reviewed the references list and ensured its completeness and accuracy.

Reviewers’ comments:

Reviewer #3

I was pleased to review PONE-D-22-11363R1 “Variation in echolocation click characteristics of finless porpoise in response to absence/presence vessel noise and day/night”. The authors ask an interesting question, and the manuscript is generally clearly written. However, I am concerned about certain aspects of the methods and the statistical analyses. As such, it is difficult to ascertain whether the interpretation of the results is in fact supported by the data.

 Thank you for reviewing our manuscript and providing your feedback. We sincerely appreciate your valuable comments and considered them during the revision process. Based on the specific points raised in your review, we primarily focused on improving the statistical analyses and refining the interpretation of the results. We kindly ask you to review the following responses, which outline the specific changes we made in light of your suggestions.

Specific comments:

P12

L240–246: “Click and click train ~, and environmental parameters,”

The authors mix information theory (i.e., model selection with an information criterion – in this case AIC) with null hypothesis testing, which is not recommended. I commend the authors to simply use GLMs (which include "study sites" as an additional explanatory variable) for all the click parameters, without testing beforehand. In addition, please note that the relative importance of model parameters and support for models is meaningless unless the authors provide a measure of the variance (or deviance) explained.

 Thank you for your comment. We took your comments into consideration and made the following revisions. Instead of performing null hypothesis testing, we opted to utilize GLMs with “study sites” as an additional explanatory variable for all click parameters. Additionally, in the Results section, we included details regarding the null deviance and residual deviance of each model. This information serves as an indicator of the amount of the variance explained.

L252–253: “...the site (factor type) was the random variable in GLMM.”

When you include "sites" as a random variable in GLMM, you cannot compare any results between study sites (because it means that you decided to regard difference between sites as a random effect, not a fixed effect). If the authors want to compare and discuss about site difference (which I think the authors do), using GLMs that include "sites" as an explanatory variable may be suitable.

 Thank you for your comment. We agree with your suggestion and made changes to the analysis methods. Specifically, we implemented GLMs to compare the study sites. In these models, we included “study site” as an additional explanatory variable in the GLMs to investigate and discuss the differences between the sites.

L253: “Salinity data~”

First appearance of "salinity" in the manuscript. Where do they come from? As there is no description about salinity data in "Environmental data" section above (and the authors do not use this data after all), please delete this sentence.

 Thank you for your comment. Regarding the salinity information, we briefly mentioned it in the current version of the manuscript in line 211–219, highlighting its use in estimating the Apparent Source Level (ASL), as stated in the Materials and Methods section. However, we acknowledge that the sentences in question were not adequately addressed in the section discussing environmental parameters influencing click characteristics. As per your comment, we recognized the potential confusion caused by these sentences and subsequently removed them from the manuscript.

P14

L284–285: “..., and environmental parameters~”

I suggest the authors to change the listing order of the parameters in the table so that background information (temp, flow, noise) comes first and data on behavioral response (i.e., click parameters) comes second. (I think listing in such order makes this table more easy-to-read for readers.)

 Thank you for your comment and suggestion. As your recommendation, we revised the order of parameters in the table 2 so that background information (temp, flow, noise) is listed first, followed by data related to behavioral response (i.e., click parameters). With this change, the caption in line 281–284 was also updated to “The summary shows environmental parameters (temperature, synthetic flow velocity, and noise level), echolocation click parameters (ASL, peak frequency, center frequency, -3 dB BW, and click duration), and click train parameters (ICI, number of clicks per train, and range from array)”. This reordering allows for a more logical and coherent presentation of the information.

P15

L303–304: “...with Residual Maximum Likelihood estimation (REML).”

This information should appear first in Methods section.

 Thank you for your comment. Since we revised our analysis method and adopted GLMs instead, the information regarding REML for GLMM is no longer necessary. Therefore, we removed that sentence from the manuscript.

L305: “GLM with gamma family~”

Information about families of distribution (as well as the link function) used for GLMs should be mentioned in Methods section, not in Results. (Note that this comment also applies to other paragraphs within this section.)

 Thank you for your comment. As you recommended, we removed all mentions of the link function information from the Results section, and included the following sentence to the Materials and Methods section, line 249–250: “ASL was analyzed using GLM with gaussian family and other parameters were analyzed using GLMs with gamma family.”

P18

L350–353: “To the best of our knowledge~”

This sentence is not a discussion; it is just stating what the author did. This should be removed. Just starting your discussion from the first section "Effects of presence~" seems enough.

 Thank you for your comment. As you suggested, we removed the sentence at L350–353 since it was not relevant to the Discussion.

L357–358: “..., which was contrary to previous findings.”

This statement is untrue because (, as the authors mentioned in Lines 361-364,) there are several studies which showed decreases of ASL in the presence of vessels. This should be toned down.

 Thank you for your valuable feedback and for bringing this issue to our attention, and we apologize for the confusion caused by lines 357 through 358 in previous manuscript. The cited paper in the previous manuscript of lines 361–364 that you pointed out was not about a decrease in ASL in the presence of vessels, but about a decrease in the emission rate of echolocation clicks. We believe that the Lombard effect, which increases the source level under noise, is the most commonly reported response under general noise conditions. In contrast, our study reveals an opposite response, where finless porpoise exhibits a decrease in sound pressure under vessel noise. To prevent confusion and follow your suggestion to tone it down, we revised this part as shown in lines 346–349 of the revised manuscript: “The phenomenon of increased vocalizations in the presence of noise is known as the Lombard effect [72], which has been observed in cetaceans [20,73] as well as in birds and bats [74], indicating a common response to noise. However, contrary to this pattern, the finless porpoises in this study exhibit a decrease in sound pressure under vessel noise.”

P19

L376–377: “... at the site with high [or low] vessel noise (St. M [or St. S])”

Although the difference in the noise level between two sites is significant (i.e., p<0.05), its effect size seems to be very small (116±1 in St. S vs. 118±2 in St. M). I think the authors should reconsider the importance and meaning of this subtle difference. In other words, “can this subtle difference in noise level (only 1 dB!) really affect behaviors of finless porpoise?”

 Thank you for your comment. Based on the feedback provided, we made revisions to the statistical analysis. As a result, the manuscript no longer includes evidence of significant differences in noise levels between the recording sites. Therefore, we removed the corresponding statement and discussion section from the manuscript.

L385: “..., there was no significant difference in the number of prey species~”

I could not find any details about sampling methods, statistics, and analyses for data regarding prey organisms. Please add this information to Methods section if the authors want to present these results here. Also, there seems a difference in the number of species between St. S (two fish species plus octopus, shrimp, squid) and St. M (eight fish species plus octopus, shrimp, squid). Please re-check.

Besides, what kind of shrimp and squid were caught? Please identify these organisms to species level (or, at least, to a higher taxonomic level; order, class, family, etc.).

 Thank you for your comments. The information on prey species was derived from previous studies and literature; it was not something we have specifically investigated in our research. We apologize for the lack of clarity on this point in the previous manuscript. As a response, we appropriately cited the relevant literature in the discussion. Furthermore, as you pointed out, regarding the number of prey species, we selected the main prey species based on the catch volume data and provided an equal number of prey species as examples in the two sites.

L386–398: “The following prey species~”

Why listing up all the species here? This section does not seem to be related to any of your discussion. Are these species diurnal or nocturnal? Which of them are important for finless porpoise? Just delete this section or add more context (if there is any importance in listing up each of prey species here).

 Thank you for your comment. The information regarding prey species is additional information that was included based on the reviewer’s suggestion. It has been revealed in previous studies that porpoises are susceptible to the influence of prey species. Therefore, we believe it is appropriate to include this information as previously recommended. Regarding the prey species, we found no significant difference between the two sites compared. And, we also considered that the discussion of the diurnal rhythm of prey species or finless porpoises deviate from the scope of this study. Therefore, we did not mention it in the manuscript and made efforts to provide concise descriptions regarding the prey species. Please refer to lines 377-385 for more details, “The prey species of finless porpoise [78] were similar at St. S and St. M, with the main prey species consisting of whitespotted conger (*Conger myriaster*), Japanese sea bass (*Lateolabrax japonicus*), octopus, shrimp, and squid [79–81]. Additionally, the following non-prey species of the finless porpoise were captured at both sites: Japanese jack mackerel (*Trachurus japonicus*), red tilefish (*Brachiostegus japonicus*), chubmackerel (*Scomber japonicu*), Japanese spanish mackerel (*Scomberomorus niphonicus*), largehead hairtall (*Trichiurus lepturus*), righteye flounder (Pleuronectidae sp.), and bastard halibut (*Paralichthys olivaceus*), pufferfish (Tetraodonidae sp.), and crab (Brachyura) [79–81]. Furthermore, there were no differences in depth or bottom sediments at both sites. Variations in ASL at both sites may likely not be due to differences in feeding time or prey species”. However, while our study did not reach definitive conclusion regarding the correlation with prey species behavior, it is an area of great interest, and we intend to further investigate and elucidate it in future research. Thank you for bringing this to our attention.

P20

L400–401: “... noise levels between the two sites were different~”

As I commented above (L376–377), the difference in noise level (i.e., 1 dB) do not seem to be great enough to cause any change in porpoise’s behavior. This point needs to be considered.

 Thank you for your comment. As we decided not to discuss the differences in noise levels between recording sites based on the previous comment, so the sentence was removed.

L414: “... by finless porpoise.”

This phrase is redundant. Please delete.

 Thank you for your comment. Following your suggestion, this phrase was removed.

L419–420: “Finless porpoises rely more on~”

Does this statement come from your observation (i.e., data presented in this manuscript) or other literature? If the latter, please provide reference.

 Thank you for your comment. This statement is our interpretation based on the results obtained in this study. To ensure clarity, we revised this sentence (in line 404–406 of the revised manuscript) to “Based on these findings, it is probable that finless porpoises rely more on acoustic information at night owing to relatively lower visual information at night”.

P21

L432, L434: “NBHF”

First appearance of "NBHF" in the manuscript. Abbreviations must be defined upon first use. Here, the authors better use "narrow-band high-frequency" instead of "NBHF" because the authors use this abbreviation only twice in the whole manuscript and I think most readers are not familiar with this abbreviation.

 Thank you for your comment. Following your suggestion, we described “narrow-band high-frequency” in full instead of using the abbreviation “NBHF”.

L436: “In this present study, ~”

This is not a discussion. This is what the authors did. Please delete.

 Thank you for your comment. Following your suggestion, we deleted this sentence.

L439–441: “... in the present study had a higher peak frequency~”

Here the authors compare four sites (two in Japan, Liao-dong wan Bay, and Taiwan strait) but only show data from two sites abroad. I think is better to delete numerical values from this sentence for simplicity. If these numerical values are important, showing numerical values for two Japanese sites might be helpful for readers to compare among four sites. In this case, providing another table (that compares values from four study sites) might be helpful.

 Thank you for your comment. Following your suggestion, we deleted numerical values for simplicity.
