# Supplementary material for: Variations in echolocation click characteristics of finless porpoise in response to day/night and absence/presence of vessel noise
Source: PLoS One. 2023 Aug 4;18(8):e0288513. doi: 10.1371/journal.pone.0288513 (PMC10403093; doi:10.1371/journal.pone.0288513)
Supplement: Attachment — Submitted filename: response to Reviewers.docx [file pone.0288513.s007.docx]

Thank you for dedicating your time reviewing and thoroughly assessing our manuscript. We sincerely appreciate your valuable feedback, which has greatly contributed to the improvement of our work. We have carefully addressed the journal’s requirements and diligently incorporated the suggestions provided by the editor. In the revised manuscript, we have used the following color scheme: black to denote the journal requirements and editor’s comments, blue to indicate our responses, and green to highlight the revised words/sentences.

-------------------------------------------------------------------------------------------------------

Academic Editor Requirements:

Do not remove the t-test and u-test descriptions from the Materials and Methods section. You are looking at significant differences using these tests in Results and Table 2. Additionally, I think it would be better to switch the order of Table2 and 3.

 Thank you for your comments. As you pointed out, we reverted the modifications that removed the descriptions of t-test and u-test descriptions from Materials and Methods section, as can be seen in line 242–245 “The environmental parameters (temperature, synthetic flow velocity, and noise level) at each site were compared using Mann–Whitney U-test. Similarly, click and click train parameters at each site were compared using Student’s t-test or Mann–Whitney U-test”. Additionally, we switched the order of Table2 and 3.

Journal Requirements:

Please review your reference list to ensure that it is complete and correct. If you have cited papers that have been retracted, please include the rationale for doing so in the manuscript text, or remove these references and replace them with relevant current references. Any changes to the reference list should be mentioned in the rebuttal letter that accompanies your revised manuscript. If you need to cite a retracted article, indicate the article’s retracted status in the References list and also include a citation and full reference for the retraction notice.

 Thank you for your comments. We carefully reviewed the reference list once again to ensure its completeness and accuracy. We took your comments into consideration and made the necessary revisions.
